# Neuronavigation in glioma resection: current applications, challenges, and clinical outcomes

**DOI:** 10.3389/fsurg.2024.1430567

**Published:** 2024-08-06

**Authors:** Albert Joseph Sulangi, Adam Husain, Haoyi Lei, Jessica Okun

**Affiliations:** ^1^ Nova Southeastern University, Dr. Kiran C. Patel College of Osteopathic Medicine—Tampa Bay Regional Campus, Clearwater, FL, United States; ^2^ University of Texas Medical Branch, Galveston, TX, United States; ^3^ Elson S. Floyd College of Medicine, Spokane, WA, United States; ^4^ Steward Medical Group, Fort Lauderdale, FL, United States

**Keywords:** neuronavigation, glioma resection, high-grade glioma, low-grade glioma, intraoperative imaging, surgical outcomes, extent of resection, gross total removal of tumor (GTR)

## Abstract

**Background:**

Glioma resection aims for maximal tumor removal while preserving neurological function. Neuronavigation systems (NS), with intraoperative imaging, have revolutionized this process through precise tumor localization and detailed anatomical navigation.

**Objective:**

To assess the efficacy and breadth of neuronavigation and intraoperative imaging in glioma resections, identify operational challenges, and provide educational insights to medical students and non-neurosurgeons regarding their practical applications.

**Methods:**

This systematic review analyzed studies from 2012 to 2023 on glioma patients undergoing surgical resection with neuronavigation, sourced from MEDLINE (PubMed), Embase, and Web of Science. A database-specific search strategy was employed, with independent reviewers screening for eligibility using Rayyan and extracting data using the Joanna Briggs Institute (JBI) tool.

**Results:**

The integration of neuronavigation systems with intraoperative imaging modalities such as iMRI, iUS, and 5-ALA significantly enhances gross total resection (GTR) rates and extent of resection (EOR). While advanced technology improves surgical outcomes, it does not universally reduce operative times, and its impact on long-term survival varies. Combinations like NS + iMRI and NS + 5-ALA + iMRI achieve higher GTR rates compared to NS alone, indicating that advanced imaging adjuncts enhance tumor resection accuracy and success. The results underscore the multifaceted nature of successful surgical outcomes.

**Conclusions:**

Integrating intraoperative imaging with neuronavigation improves glioma resection. Ongoing research is vital to refine technology, enhance accuracy, reduce costs, and improve training, considering various factors impacting patient survival.

## 1 Introduction

### 1.1 Overview of gliomas

Glioma is the predominant type of central nervous system neoplasm arising from glial cells, characterized by diffuse infiltration into the surrounding brain tissue, with glioblastoma being highly malignant and pilocytic astrocytomas representing the least malignant form (1). The Central Brain Tumor Registry of the United States (CBTRUS) data on gliomas constitute 26.3% of all brain tumors, with glioblastoma being the most common malignant histopathology, accounting for 14.2% of all tumors and 50.9% of malignant tumors (2). Surgical resection plays a pivotal role in the management of malignant gliomas, representing the gold standard therapy aimed at achieving maximum possible tumor removal (3). While research continues in the pursuit of a cure, current treatment protocols emphasize extending survival and improving quality of life. The development of surgery for glial tumors has seen significant progress. In the late 19th century, glioma surgery faced challenges with limited successful cases, but pioneering surgeons like Cushing navigated infiltrating growths (4), shaping the foundational period of neurosurgery with diverse surgical goals. The groundbreaking surgery performed by Bennett and Godlee in the early 20th century was pivotal in the evolution of neurosurgery, particularly in the treatment of gliomas (5). By daring to operate on a brain tumor without the aid of modern tools or imaging technologies, their work not only highlighted the crucial role of scientific knowledge and technological advancements in surgery but also paved the way for future innovations (5). Their success under these primitive conditions underscored the importance of accurate tumor localization prior to surgery, fundamentally enhancing the surgical approach to glioma treatment and demonstrating the transformative impact of medical innovations on patient outcomes (5). Over the last 50 years, craniotomy outcomes for malignant astrocytomas improved, with the operating microscope and advanced technologies contributing to radical resections and refined glioma excision (5). The evolution of stereotactic techniques further transformed glioma surgery, offering surgical guidance and a complementary approach to conventional methods (5).

### 1.2 Evolution of neuronavigation

Neuronavigation, likened to a GPS for neurosurgeons, is an advanced technological application used in neurosurgery to enhance the precision of interventions involving brain structures (6). The historical development of neuronavigation traces back to Sir Victor Horsely and engineer Robert Clarke, who conceptualized a three-dimensional coordinate system for the brain, giving rise to the Horsley-Clarke frame in the late 19th century (7). Early stereotactic devices faced reliability issues until the development of a radiology-based system by Spiegel and Wycis in Philadelphia four decades later (8). The advent of computed tomography (CT) in 1971, pioneered by Godfrey Hounsfield, revolutionized neuronavigation, leading neurosurgeons to adapt stereotactic frames for CT scans and later for magnetic resonance imaging (MRI) (9).

### 1.3 Principles and techniques of neuronavigation

**Figure 1:**
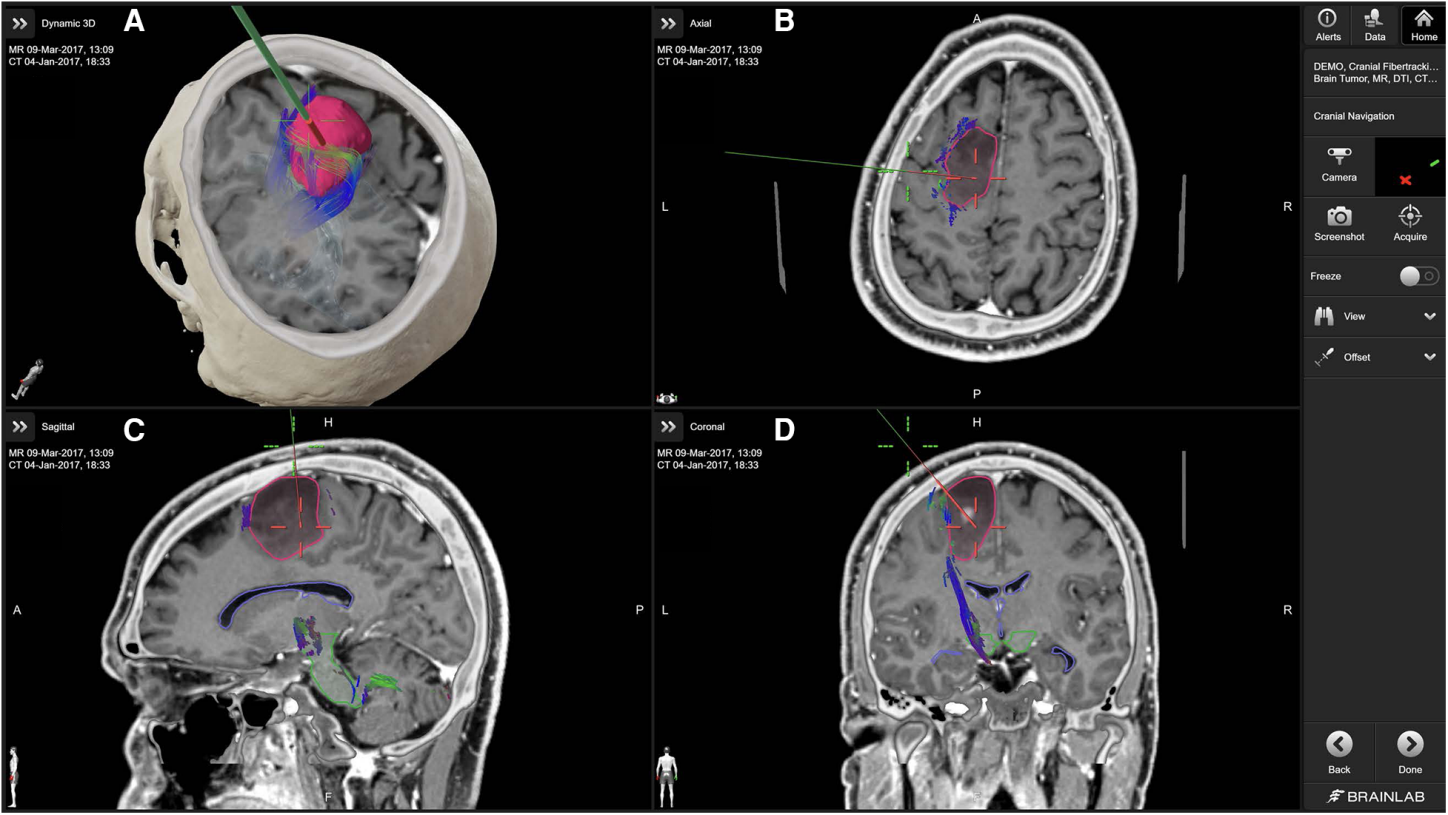
Cranial navigation with brainlab cranial navigation (10). The primary panel (**A**) displays a 3D reconstruction combining MRI and CT data, with the tumor highlighted in red and associated fiber tracts in various colors. The axial (**B**), sagittal (**C**), and coronal (**D**) views provide detailed cross-sectional images of the brain, aiding in the precise localization of the tumor and its relationship to critical white matter tracts. The green navigation probe illustrates the planned surgical approach.

**Figure 2:**
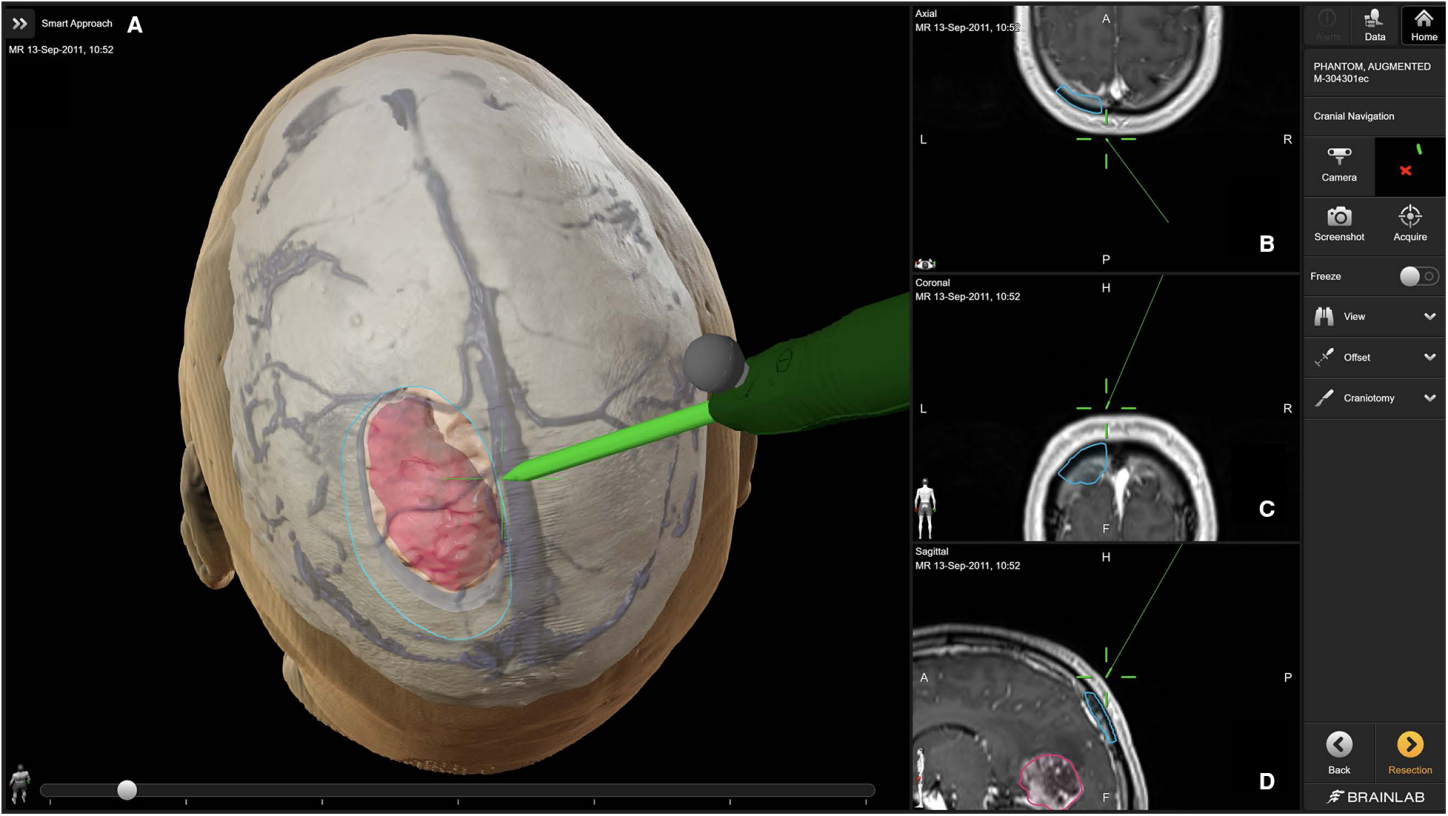
Access and craniotomy planning with brainlab cranial navigation (11). The primary panel (**A**) showcases a 3D reconstruction of the patient's head, detailing the planned craniotomy (outlined in blue) and the tumor (highlighted in pink). The navigation probe (green) illustrates the intended surgical approach. The right panels display axial (**B**), coronal (**C**), and sagittal (**D**) MRI slices, providing precise anatomical context for the planned surgical route.

Neuronavigation integrates preoperative imaging data such as CT and MRI to create a three-dimensional map of the patient's brain (Figure 1). This map is then used during surgery to guide the neurosurgeon to the exact location of interest, such as a tumor or other pathology (Figure 2). The process begins with the acquisition of high-resolution images, where fiducial markers may be placed on the patient's scalp to serve as reference points (12, 13). These markers are crucial for the registration phase, which is the process of aligning the preoperative images with the actual position of the patient's head during surgery (12). There are various methods of registration, including using adhesive markers, bone fiducials, or surface scanning of the head to match the images with physical landmarks (12).

Once registration is completed, the neuronavigation system can track surgical instruments in relation to the patient’s brain anatomy displayed on a monitor (13). This tracking is facilitated through either optical systems, which require a direct line of sight to the instruments, or electromagnetic systems, which do not require line-of-sight and allow more flexibility in instrument handling (12, 14, 15).

However, neuronavigation systems are not without limitations. They depend heavily on the accuracy of preoperative images and the registration process (13, 14). Any movement of the patient or error in initial marker placement can lead to inaccuracies (13). Additionally, changes in the brain that occur during surgery, such as brain shift due to swelling or removal of tissue, can also affect the accuracy of neuronavigation (12, 16). This limitation necessitates the use of additional intraoperative imaging technologies such as intraoperative CT, MRI, and ultrasonography. These technologies provide real-time images of the brain, allowing the neuronavigation system to update its maps to reflect the current state of the brain (12).

This systematic review seeks to synthesize recent studies on the integration of neuronavigation with intraoperative imaging modalities to optimize glioma resection strategies. It focuses on assessing the precision and effectiveness of these techniques, while also identifying the challenges and limitations encountered in their implementation. The primary goal of this review is to provide medical students and non-neurosurgeons with a comprehensive understanding of neuronavigation and intraoperative imaging technologies used in glioma surgery. By doing so, it aims to equip future neurosurgeons with the knowledge and insights needed to apply these sophisticated technologies effectively in glioma resections.

## 2 Methods

The proposed systematic review will be conducted in accordance with the JBI methodology for systematic reviews of qualitative evidence (17). In this systematic review, the population is defined as patients diagnosed with gliomas undergoing surgical resection. The interventions under review include neuronavigation and advanced intraoperative imaging techniques. The comparators consist of neuronavigation and various imaging modalities. The primary outcomes assessed are gross total resection rates (GTR), extent of resection (EOR), and survival rates. The analysis includes studies of randomized controlled trials, clinical trials, and observational studies.

### 2.1 Review questions

What is the comprehensive impact of neuronavigation techniques on the outcomes of glioma resection, considering their diverse applications, accuracy, challenges in implementation, and integration with advanced imaging modalities?

Spectrum of Neuronavigation Techniques:

1. What is the diversity of neuronavigation techniques employed in glioma resection?
  • How do these techniques contribute to the current clinical practice, and what variations exist in their applications?

Accuracy and Efficacy of Neuronavigation:

2. To what extent do neuronavigation techniques enhance the accuracy of glioma resection, considering parameters such as the extent of tumor removal, functional preservation, and postoperative neurological outcomes?
  • What is the qualitative and quantitative synthesis of available studies regarding the impact of neuronavigation on glioma resection outcomes?

Challenges and Limitations in Neuronavigation Implementation:

3. What challenges and limitations are associated with the implementation of neuronavigation in glioma surgery?
  • How do these challenges affect the effectiveness of neuronavigation-guided procedures?

Integration of Advanced Imaging Modalities with Neuronavigation:

4. How is advanced imaging integrated with neuronavigation to optimize strategies for glioma resection?
  • What evidence exists regarding the synergy between neuronavigation and advanced imaging modalities in improving surgical planning, enhancing tumor targeting, and preserving critical brain functions?

### 2.2 Inclusion criteria

*Subjects of the study:* Patients diagnosed with gliomas undergoing surgical resection.

*Phenomena of interest:* Studies investigating usefulness of neuronavigation techniques in glioma resection, with outcome data.

*Types of Studies:* Randomized controlled trials, clinical trials, and observational studies reporting primary data and outcomes on the use of neuronavigation, published in English, between 2012 and 2023, with full text accessible for review.

The inclusion criterion of limiting studies to those published in English is justified to maintain consistency in language comprehension among researchers and readers, ensuring effective communication and interpretation of findings. Limiting the inclusion criteria to studies published between 2012 and 2023 ensures the relevance of the systematic review by focusing on recent advancements and findings in the field while excluding outdated information.

### 2.3 Exclusion criteria

*Subjects of the Study:* Studies focusing on other types of cancers or neurological conditions without a clear focus on the specified gliomas.

*Types of Studies:* Nonclinical studies, reviews, editorials, commentaries, and case reports. Abstracts, conference proceedings, and unpublished data were also excluded.

### 2.4 Search strategy

The search strategy will aim to locate published studies. A three-step search strategy will be utilized in this review. First, an initial limited search of MEDLINE (PubMed) was undertaken to identify articles on the topic (see Supplementary Table S2.1). The text words contained in the titles and abstracts of relevant articles, and the MeSH terms used to describe the articles were used to develop a full search strategy for MEDLINE (PubMed), Embase, and Web of Science (see Supplementary Data: Search Strategy). The search strategy, including all identified text words and MeSH terms, will be adapted for each included database. The reference list of all included sources of evidence will be screened for additional studies.

### 2.5 Study selection

**Figure 3:**
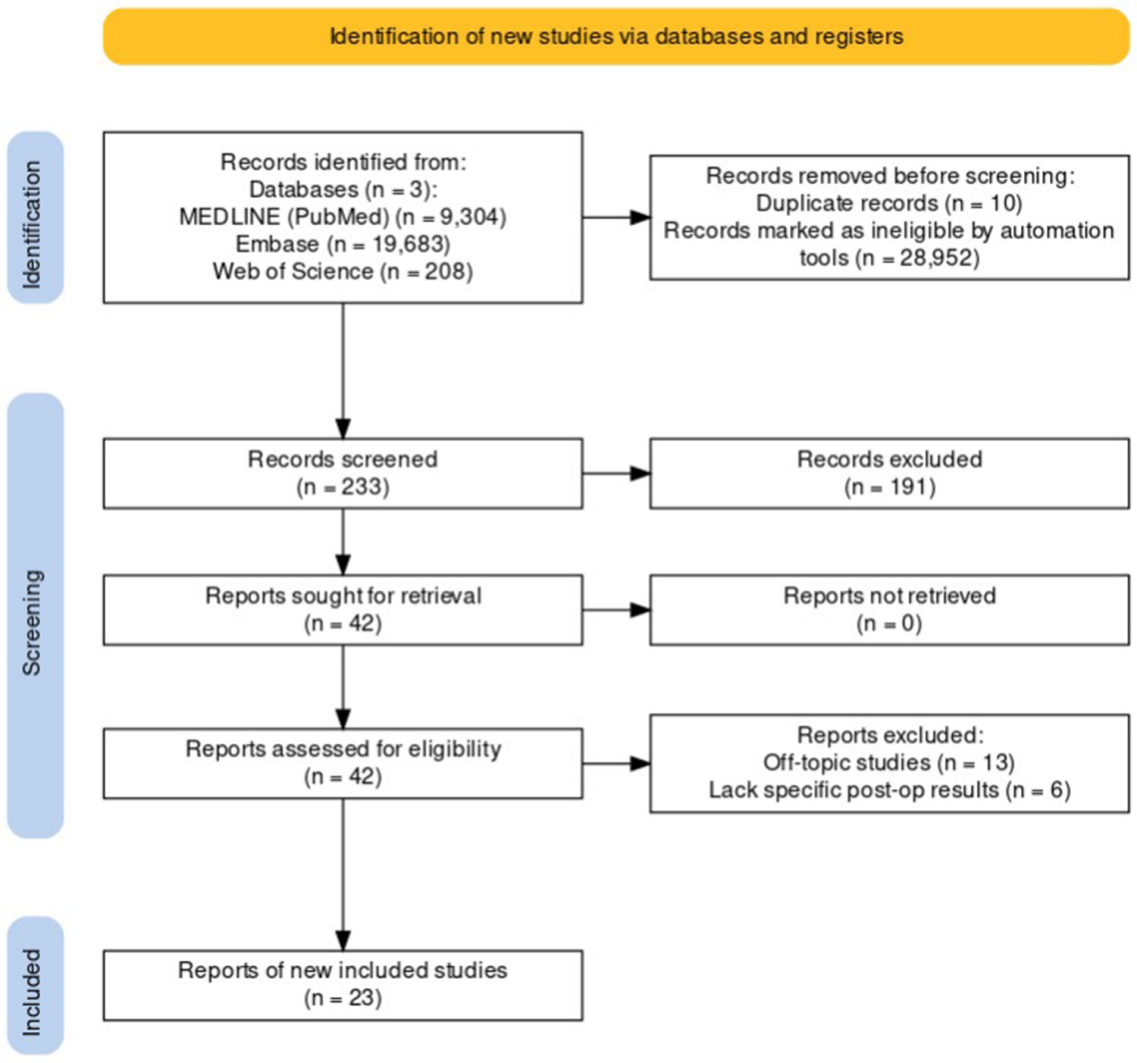
PRISMA Flow Diagram for the Selection Process of Studies on Neuronavigation in Glioma Resection.

Following the search, all identified citations will be collated and uploaded into Rayyan (18) and duplicates will be removed. Following a pilot test, three independent reviewers will screen the titles and abstracts for assessment against the inclusion criteria for the review. Potentially relevant studies will be retrieved in full, and their citation details imported into the JBI System for the Unified Management, Assessment and Review of Information (JBI SUMARI) (17) (JBI, Adelaide, Australia). Two or more independent reviewers will assess the full text of selected citations in detail against the inclusion criteria. Reasons for exclusion of papers at full text that do not meet the inclusion criteria will be recorded and reported in the systematic review (see Supplementary Table S2.2). Any disagreements that arise between the reviewers at each stage of the selection process will be resolved through discussion, or with an additional reviewer/s. The results of the search and the study inclusion process will be reported in full in the final systematic review and presented in a Preferred Reporting Items for Systematic Reviews and Meta-analyses (PRISMA) flow diagram (Figure 3) (19, 20).

### 2.6 Data extraction

Data will be extracted from studies included in the review by two independent reviewers using the standardized JBI data extraction tool (17). The data extracted will include specific details about the patient characteristics, spectrum of neuronavigation techniques, accuracy and efficacy of neuronavigation, challenges and limitations in neuronavigation limitation, and integration of advanced imaging modalities with neuronavigation (see Supplementary Table S2.3). Any disagreements that arise between the reviewers will be resolved through discussion or with a third reviewer. Authors of papers will be contacted to request missing or additional data, where required.

### 2.7 Data synthesis

Qualitative research findings will, where possible, be pooled with the meta-aggregation approach. This will involve the aggregation or synthesis of findings to generate a set of statements that represent that aggregation, through assembling the findings and categorizing these findings based on similarity in meaning. These categories will then be subjected to a synthesis to produce a single comprehensive set of synthesized findings. Where textual pooling is not possible the findings will be presented in narrative form. Only unequivocal and credible findings will be included in the synthesis (see Supplementary Table S2.4).

## 3 Results

### 3.1 Patient characteristics

This systematic review encompasses an analysis of studies involving 1,521 patients undergoing glioma resection, with study sizes ranging from 7 to 145 participants. The patient demographics generally include a mixture of males and females, with a higher incidence among males. The age distribution spans mid to late adulthood, with the average ages of cohorts ranging from 39 to 66 years old.

Regarding the glioma grades, the studies reveal varied distributions. Some exclusively report high-grade gliomas, classified as WHO Grade III and IV, while others present a blend of both high-grade and low-grade gliomas, the latter comprising WHO Grade I and II. This variation is observed across different study and control groups, with some details not specified in certain reports. An overview of patient characteristics across the included studies is provided in Supplementary Table S2.5.

### 3.2 Impact of neuronavigation (NS) and advanced intraoperative imaging techniques (AIIT) on selected neurosurgical metrics and outcomes

Table 1 presents the overview of studies on the impact of NS and AIIT on operative time (OT), GTR and EOR. The gross total resection rate indicates the percentage of patients in whom the entirety of the targeted tissue was removed. The extent of resection measures how much of the tumor is removed. Research generally shows that advanced imaging and enhancement technologies, when integrated with neuronavigation systems, significantly improve neurosurgical outcomes, particularly in GTR and EOR. Zhang et al., Fujii et al., and Lu et al. all found that intraoperative MRI substantially increases GTR and EOR, enhancing surgical precision (22, 24, 25). Incekara et al. noted a modest boost in EOR from using intraoperative ultrasound (iUS) with neuronavigation (28). Despite these advantages, the addition of these technologies did not consistently reduce operative times, as seen in the findings of Fujii et al. and Incekara et al., indicating that while these tools improve the quality of surgery, they do not necessarily expedite the process (24, 28). Coburger et al. and Eyüpoglu et al. achieved nearly perfect GTR and EOR by combining 5-aminolevulinic acid (5-ALA) with iMRI and neuronavigation, demonstrating significant gains in resection quality (37, 38). Additionally, Wang et al. observed that sodium fluorescein not only improved GTR but also reduced operative times, contributing to greater surgical efficiency (41). Picht et al. confirmed that navigated transcranial magnetic stimulation (nTMS) with intraoperative monitoring (IOM) enhances tumor targeting and resection (42). These findings underline the critical impact of combining these technologies in elevating the standards of neurosurgical practice.

**Table 1:** Overview of studies on NS and AIIT and their impact on GTR and EOR.

Source	NS	Cohort	AIIT	Number of patients	OT	GTR	EOR
Kubben et al. (21)	Medtronic StealthStation	SG	NS + iMRI	7	NR	NR	13% (median)^a^
		CG	NS	7	NR	NR	6.5% (median)^a^
Zhang et al. (22)	Brainlab iPlan 2.6	SG	NS + iMRI	112	NR	69.60%	95.50% (mean)
		CG	NS	86	NR	47.70%	89.85% (mean)
Chen et al. (23)	Brainlab iPlan 2.6	CG	NS + iMRI	51	390 min	NR	96% (median)
		SG	NS	22	378 min	NR	84% (median)
Fujii et al. (24)	Brainlab Curve Dual Display™	SG	NS + iMRI	11	465.8 min	73%	NR
		CG	NS	11	483.6 min	18%	NR
Lu et al. (25)	Medtronic StealthStation TRIA i7	SG	NS + iMRI	20	355.85 min	96.55%	NR
		CG	NS	20	302.45 min	87.70%	NR
Zhang et al. (26)	Brainlab iPlan Cranial 3.0		NS + iMRI + 3D 1H-MRS	15	NR	86.67%	NR
Akay et al. (27)	Medtronic StealthStation S7		NS + iMRI (DTI)	18	NR	50%	NR
Incekara et al. (28)	Brainlab	SG	NS + iUS	23	177 min	35%	97% (median)
		CG	NS	24	179 min	8%	95% (median)
De Witt Hamer et al. (29)	Brainlab iPlan 3.0	Senior Team	iUS	56	NR	41%	66% (median)
		Junior Team	NS	52	NR	73%	92% (median)
Unsgård et al. (30)	SonoWand Invite		NS + iUS	15	NR	NR	NR
Hou et al. (31)	Brainlab iPlan 3.0		NS + iUS + iMRI	40	270 min	72.50%	95.43% (mean)
Chan et al. (32)	Name not reported		NS + iUS + 5-ALA	16	290.4 min	56.25%	NR
Cordova et al. (33)	Name not reported		NS + 5-ALA	30	NR	NR	94.30% (median)
Della Puppa et al. (34)	Name not reported		NS + 5-ALA	94	NR	93%	NR
Bettag et al. (35)	Brainlab VectorVision Sky		NS + 5-ALA	12	NR	100%	244.70% (mean)
Hauser et al. (36)	Medtronic StealthStation S7		NS + 5-ALA + iMRI	11	NR	82%	NR
Coburger et al. (37)	Brainlab iPlan 3.0	SG	NS + 5-ALA + iMRI	33	NR	100%	99.70% (mean)
		CG	NS + iMRI	33	NR	82%	97.40% (mean)
Eyüpoglu et al. (38)	Brainlab VectorVision	SG	NS + 5-ALA + iMRI	30	NR	100%	136% (median)
		CG	NS + iMRI	75	NR	100%	NR
Schatlo et al. (39)	Name not reported	SG	NS + 5-ALA + iMRI	145	NR	45%	NR
		CG	NS + 5-ALA	55	NR	30%	NR
Margetis et al. (40)	Brainlab		NS + Indigo Carmine dye	10	NR	NR	97.1% (mean)
Wang et al. (41)	Name not reported	SG	NS + Sodium Fluo-rescein	60	229.11 min	86.67%	NR
		CG	NS	60	285.13 min	60%	NR
Picht et al. (42)	Brainlab iPlan 2.0	SG	NS + nTMS + IOM	93	219 min	61%	85.40% (mean)
		CG	IOM ± NS	34	228 min	45%	75.90% (mean)
Krieg et al. (43)	Brainlab	SG	NS + nTMS + PET ± 5-ALA	70	201 min	NR	34.3% (rate)^a^
		CG	NS + PET ± 5-ALA	70	208 min	NR	54.3% (rate)^a^

^a^ Residual tumor volume.

In Table 2, several studies have demonstrated a clear link between the effectiveness of neurosurgical interventions, particularly in achieving high GTR and EOR, and improved outcomes in overall survival (OS) and progression-free survival (PFS). Zhang et al. and Coburger et al. both found that integrating advanced technologies like intraoperative MRI (iMRI) significantly enhances GTR and EOR, leading to notably better OS and PFS (22, 37). Similarly, Eyüpoglu et al. and Schatlo et al. reported that near-perfect resection rates substantially extend patient survival, emphasizing the critical importance of complete tumor removal (38, 39). Wang et al. highlighted that the use of sodium fluorescein improves visualization and resection outcomes, correlating with better survival metrics (41). In contrast, Fujii et al., Lu et al., Akay et al., and Hauser et al. underscore a gap in data linking high GTR rates directly to survival benefits, suggesting that while effective resection is crucial, it may not be the sole factor influencing long-term patient survival (24, 25, 27, 36). Krieg et al. noted that nTMS led to varied residual tumor rates, with lower residuals associated with longer survival, reinforcing that more thorough resections can lead to improved survival outcomes (43). In summary, while advanced imaging and neuronavigation significantly enhance neurosurgical precision and patient outcomes, particularly in achieving high GTR and EOR which are linked to improved survival rates, they do not universally expedite operative times and their impact on long-term survival can vary, indicating that successful surgical outcomes involve a complex interplay of factors beyond just technological integration. A comprehensive overview of neuronavigation and advanced imaging with neurosurgical metrics and outcomes is provided in Supplementary Table S2.6.

**Table 2:** Overview of studies on NS and AIIT and their impact on OS, and PFS.

Source	NS	Cohort	AIIT	OS	PFS
Kubben et al. (21)	Medtronic StealthStation	SG	NS + iMRI	13 months (median)	NR
		CG	NS	15.5 months (median)	NR
Zhang et al. (22)	Brainlab iPlan 2.6	SG	NS + iMRI	19.6 months (median)	12.5 months (median)
		CG	NS	13 months (median)	6.6 months (median)
Chen et al. (23)	Brainlab iPlan 2.6	CG	NS + iMRI	28 months (median)	18 months (median)
		SG	NS	18 months (median)	15 months (median)
Fujii et al. (24)	Brainlab Curve Dual Display™	SG	NS + iMRI	NR	NR
		CG	NS	NR	NR
Lu et al. (25)	Medtronic StealthStation TRIA i7	SG	NS + iMRI	NR	NR
		CG	NS	NR	NR
Zhang et al. (26)	Brainlab iPlan Cranial 3.0		NS + iMRI + 3D 1H-MRS	NR	12 months
Akay et al. (27)	Medtronic StealthStation S7		NS + iMRI (DTI)	15.3 months (mean)	36.4% (PFS at 6 months)
Incekara et al. (28)	Brainlab	SG	NS + iUS	12.4 months (median)	7.5 months (median)
		CG	NS	12.2 months (median)	7.7 months (median)
De Witt Hamer et al. (29)	Brainlab iPlan 3.0	Senior Team	iUS	NR	NR
		Junior Team	NS	NR	NR
Unsgård et al. (30)	SonoWand Invite		NS + iUS	10.9 months (median)	42% (PFS at 6 months)
Hou et al. (31)	Brainlab iPlan 3.0		NS + iUS + iMRI	NR	NR
Chan et al. (32)	Name not reported		NS + iUS + 5-ALA	NR	NR
Cordova et al. (33)	Name not reported		NS + 5-ALA	81% at 6 months, 52% at 9 months, 39% at 12 months	45% at 6 months, 29% at 9 months, 23% at 12 months
Della Puppa et al. (34)	Name not reported		NS + 5-ALA	NR	NR
Bettag et al. (35)	Brainlab VectorVision Sky		NS + 5-ALA	NR	NR
Hauser et al. (36)	Medtronic StealthStation S7		NS + 5-ALA + iMRI	15.3 months (mean)	36.4% (PFS at 6 months)
Coburger et al. (37)	Brainlab iPlan 3.0	SG	NS + 5-ALA + iMRI	18 months (median)	6 months (median)
		CG	NS + iMRI	17 months (median)	6 months (median)
Eyüpoglu et al. (38)	Brainlab VectorVision	SG	NS + 5-ALA + iMRI	18.5 months (median)	NR
		CG	NS + iMRI	14 months (median)	NR
Schatlo et al. (39)	Name not reported	SG	NS + 5-ALA + iMRI	17.9 months (median)	10.6 months (median)
		CG	NS + 5-ALA	13.8 months (median)	7 months (median)
Margetis et al. (40)	Brainlab		NS + Indigo Carmine dye	NR	NR
Wang et al. (41)	Name not reported	SG	NS + Sodium Fluo-rescein	11.5 months (median)	9.5 months (median)
		CG	NS	9.6 months (median)	7.5 months (median)
Picht et al. (42)	Brainlab iPlan 2.0	SG	NS + nTMS + IOM	NR	NR
		CG	IOM ± NS	NR	NR
Krieg et al. (43)	Brainlab	SG	NS + nTMS + PET ± 5-ALA	15.7 months (mean)	NR
		CG	NS + PET ± 5-ALA	11.9 months (mean)	NR

## 4 Discussion

Advanced imaging and neuronavigation technologies significantly improved GTR and EOR, with notable enhancements from iMRI, 5-ALA, and sodium fluorescein. Despite these improvements, operative times were not consistently reduced, and the impact on long-term survival varied. Studies showed better OS and PFS with higher GTR and EOR, emphasizing the critical importance of complete tumor removal.

### 4.1 Analysis of effectiveness of NS and AIIT in achieving GTR

**Figure 4:**
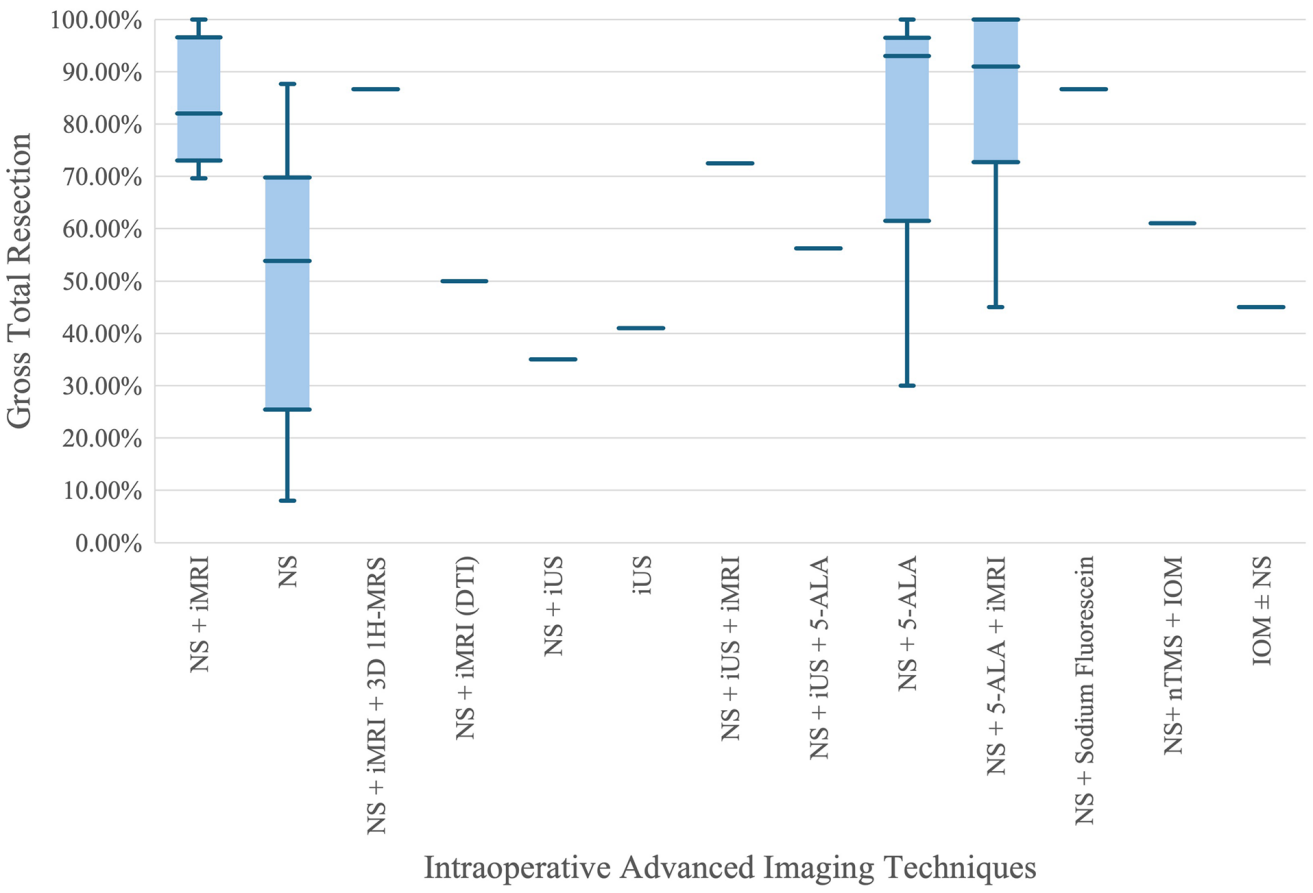
Comparison of gross total resection percentages across advanced intraoperative imaging techniques.

Figure 4 presents a comparative analysis of the effectiveness of various advanced intraoperative imaging techniques in achieving GTR. The X-axis lists different combinations of surgical navigation systems (NS) and intraoperative imaging techniques, such as intraoperative MRI (iMRI), intraoperative ultrasound (iUS), 5-aminolevulinic acid (5-ALA), and others. The Y-axis shows the GTR percentages, providing a measure of the completeness of tumor resection achieved by each technique.

From the plot, it is evident that combinations involving iMRI, such as NS + iMRI and NS + 5-ALA + iMRI, exhibit higher median GTR percentages compared to NS alone. Specifically, NS + iMRI has a median GTR percentage close to 90%, with a relatively narrow interquartile range (IQR), indicating consistent high performance. On the other hand, NS alone shows a significantly lower median GTR percentage around 50%, with a much wider IQR and whiskers extending down to nearly 10%, reflecting greater variability and lower overall effectiveness. This suggests that the integration of iMRI with NS enhances the accuracy and success of tumor resection.

Another key observation is the performance of NS + 5-ALA and NS + 5-ALA + iMRI, both of which show high median GTR percentages. The former approaches a median close to 100%, indicating that 5-ALA, a fluorescence-guided technique, greatly improves resection outcomes. The combination of NS + 5-ALA + iMRI also shows high median and consistent results, reinforcing the benefit of using multiple advanced imaging techniques. Conversely, techniques like NS + iUS and iUS alone show lower and more varied GTR percentages, suggesting that while ultrasound provides some benefit, it is less effective than iMRI or 5-ALA when used alone. Overall, the plot highlights the significant advantage of using advanced imaging adjuncts, particularly iMRI and 5-ALA, in achieving higher and more consistent GTR rates in neurosurgical procedures.

### 4.2 Relationship between GTR and OS

**Figure 5:**
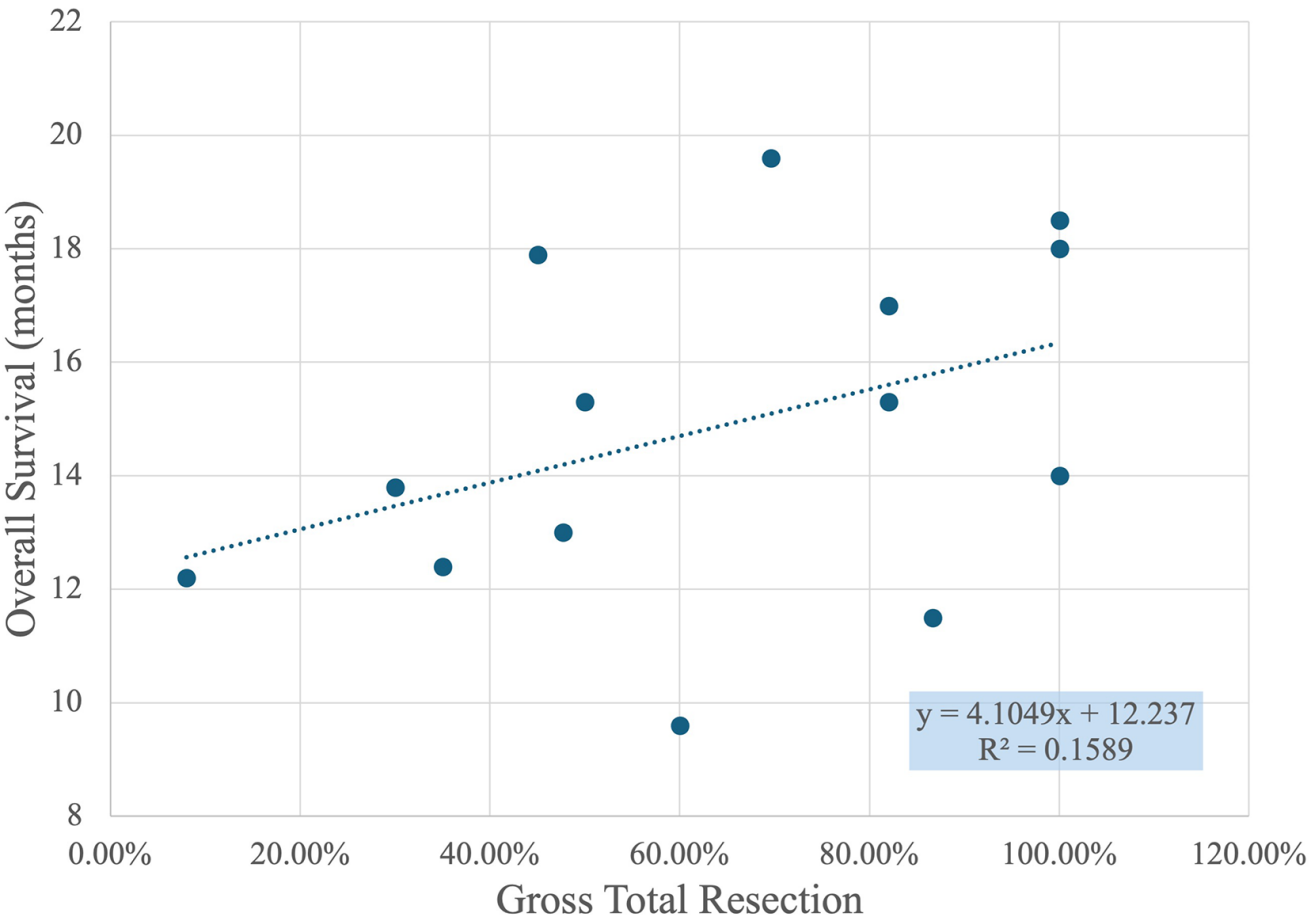
Correlation between gross total resection percentage and overall survival in months.

Figure 5 shows a scatter plot with a linear regression line that illustrates the relationship between GTR percentage and overall survival in months. Each point on the plot represents a data set where GTR percentages are compared to the corresponding patient survival times. The X-axis displays the GTR percentage, ranging from 0% to 120%, while the Y-axis shows overall survival, measured in months, ranging from approximately 8 to 22 months.

The trend line, defined by the equation y = 4.1049x + 12.237, suggests a positive correlation between GTR percentage and overall survival. Specifically, the slope of 4.1049 indicates that for every 10% increase in GTR, the overall survival time increases by approximately 0.4 months. However, the R-squared value of 0.1589 shows that the fit of this linear model is relatively weak, implying that GTR percentage alone does not fully explain the variability in overall survival times. Other factors not captured in this graph may also significantly influence survival outcomes.

Key observations from the scatter plot include the clustering of points around higher GTR percentages, especially near 100%, indicating frequent high resection success. Despite the weak correlation, there is a general trend where higher GTR percentages correspond to longer survival times. This suggests that achieving a higher GTR is generally associated with better patient outcomes, although the variability indicates that it is not the sole determinant of survival. Further research could investigate additional variables that contribute to patient survival, such as the use of different surgical techniques, patient health conditions, and post-operative care.

### 4.3 Neuronavigation with iMRI

Intraoperative MRI provides real-time, high-resolution images that help detect residual tumor tissue during the surgery. iMRI is used alongside neuronavigation to overcome some of the limitations of neuronavigation alone. One such limitation is the occurrence of brain shift, which refers to the movement of brain structures during surgery that can render preoperative images inaccurate as the surgery progresses (24). iMRI helps in updating the neuronavigation data to reflect these changes, thus maintaining the accuracy of the surgical approach throughout the procedure (24). By accurately identifying the boundaries of the tumor and avoiding eloquent brain areas, the combined use of iMRI and neuronavigation minimizes neurological deficits post-surgery, thus improving survival outcomes and preserving the patient's quality of life (21, 22). Additionally, iMRI acts as a quality control tool during surgery, allowing surgeons to verify the extent of tumor removal before concluding the procedure. This immediate feedback loop significantly reduces the likelihood of leaving behind residual tumor tissue, which could necessitate additional treatments or surgeries (24).

The combination of iMRI with neuronavigation is particularly beneficial in achieving supra-total resections, which aim to remove not just the visible tumor but also the peripheral areas that might harbor microscopic disease. This advanced approach is crucial, especially when the tumor is in eloquent areas of the brain, presenting significant surgical challenges. In such cases, the integration of iMRI with other techniques such as DTI-based neuronavigation and direct cortical stimulation during awake craniotomies helps preserve essential brain functions (27). Diffusion tensor imaging is a sophisticated MRI technique that provides detailed maps of the brain's white matter tracts, particularly in eloquent brain areas, where the risk of damaging critical neural pathways is significant (27).

Moreover, the integration of 1H-MRS (proton magnetic resonance spectroscopy) into neuronavigation systems exemplifies a shift towards multimodal approaches that not only rely on anatomical images but also incorporate metabolic information (26). This enhancement enables surgeons to delineate the metabolic boundaries of the tumor more accurately, which often extend beyond what is visible on traditional MRIs (26). The 1H-MRS provides a detailed chemical profile of the tumor, offering insights into tumor cell metabolism and infiltration, thus aiding in more precise surgical planning and execution (26).

### 4.4 Neuronavigation with iUS

The iUS with a standard neuronavigation system enables real-time overlay of ultrasound images on preoperative MRI scans (28). This integration offers a time- and cost-effective alternative to iMRI, enabling surgeons to frequently check for residual tumor during surgery without prolonging its duration (28). However, interpreting iUS images can be challenging due to their lower clarity and detail compared to the more detailed visual information provided by iMRI (28). De Witt Hamer et al. and Unsgård et al. highlight iUS's utility in surgical settings, particularly in functional mapping and identifying vital white matter pathways during resection (29, 30). Further enhancing iUS utility, the novel acoustic coupling fluid (ACF) developed by Unsgård et al. improves ultrasound image quality by reducing artifacts that can obscure small tumor remnants, thus providing clearer guidance for complete tumor resection (30). Moreover, Hou et al. discuss the benefits of combining iUS's capability to provide nearly real-time imaging for ongoing resection control and iMRI's high-quality imaging for final assessment (31). Conjointly, these technologies ensure a thorough monitoring of the resection process, allowing for adjustments based on immediate imaging feedback.

### 4.5 Neuronavigation with contrast media

Enhancing neuronavigation with contrast media like 5-ALA, indigo carmine, and sodium fluorescein improves surgical outcomes by providing immediate visual aids that clearly highlight tumor margins. 5-ALA, a precursor in heme biosynthesis, is metabolized into the fluorescent compound protoporphyrin IX (PpIX) in tumor cells (33). This conversion aids surgeons in visualizing malignant tissues during surgery, facilitating the real-time identification of tumor boundaries and providing a clearer distinction between malignant and healthy tissues (33). The practical application of 5-ALA in neuronavigation involves administering it preoperatively, typically at a dose of 20 mg/kg a few hours before surgery. During the operation, a specialized surgical microscope adapted for fluorescence excitation is used, allowing the surgeon to switch between normal and violet-blue light, under which the PpIX fluoresces, illuminating the tumor tissues. Neuronavigation systems, preloaded with preoperative MRI data, guide the resection process by aligning the intraoperative fluorescent visualization with the neuroanatomical data, ensuring precise microsurgical removal of the glioma, especially along the contrast-enhancing margins (34, 35).

Similarly, sodium fluorescein enhances the visibility of tumor tissues during neuronavigation-guided microsurgery (41). It is administered intravenously and accumulates in the tumor tissues, causing them to fluoresce under specialized light. This fluorescence assists surgeons in distinguishing tumor tissue from healthy brain tissue, especially in high-grade gliomas with ambiguous boundaries (41). Lastly, indigo carmine dye is used in a novel approach to overcome the limitations associated with traditional neuronavigation, which can be compromised by intraoperative brain shift (40). Developed by Margetis et al. (2015), the technique involves the stereotactic injection of indigo carmine into the deep tumor margins before the craniotomy and dura are opened (40). This preoperative marking ensures that the tumor margins are visually identifiable throughout the surgery, regardless of any brain shift. The dye is injected through small bur holes using a spinal needle, which is registered to the stereotactic system to precisely target the most challenging tumor margins identified from preoperative MRI scans (40). This method not only marks the perimeter but also the deep aspects of the tumor, facilitating a more complete resection (40).

### 4.6 Neuronavigation with 5-ALA and iMRI

Integrating 5-ALA fluorescence with iMRI within a neuronavigation framework allows for a more refined and aggressive approach to tumor removal (37). The process typically begins with the administration of 5-ALA preoperatively, which is absorbed by the tumor cells and converted into the fluorescent marker visible during surgery (36, 37, 39). During the operation, neuronavigation is used to guide the resection process based on preoperative MRI data (36, 37, 39). As the surgery progresses, 5-ALA helps in identifying and resecting fluorescent tumor tissues. After this phase, an iMRI scan is performed to check for any residual tumor mass (36, 37, 39). If the iMRI shows remaining contrast-enhancing areas, the surgeon can return to those specific sites and perform additional resection under the guidance of both the neuronavigation system and the fluorescence visualization (36, 37, 39). This approach ensures a more thorough resection, potentially extending the patient's progression-free and overall survival rates (36, 37, 39).

This method's efficacy lies in its ability to address the limitations of each imaging modality alone. While 5-ALA provides high specificity in detecting malignant cells, it may not always delineate the full extent of the tumor due to its reliance on cellular metabolism which might not highlight all cancerous cells (38). iMRI compensates for this by providing a structural view of the brain, revealing any remaining tumor masses that were not fluorescent (38). The sequential use of fluorescence to maximize tumor identification followed by iMRI to confirm the completeness of resection exemplifies a strategic approach to glioma surgery, maximizing safety and efficacy (38).

### 4.7 Neuronavigation with nTMS, IOM, and PET

Navigated transcranial magnetic stimulation (nTMS) along with modalities like intraoperative neuromonitoring (IOM), and preoperative positron emission tomography (PET) are integrated into neuronavigation systems to enhance the precision and safety of glioma resections. The use of nTMS, in particular, has been shown to modify surgical plans in a significant percentage of cases, directly impacting the EOR and ultimately the survival rates of patients with gliomas located near or within motor eloquent areas (42, 43). The integration process begins with preoperative planning and mapping: high-resolution MRI scans detail the brain's anatomy and the tumor's location relative to critical motor areas (42). The nTMS system then utilizes these MRI images to guide a magnetic coil over the scalp, targeting and stimulating motor areas. The responses, crucial for motor control, are recorded to create motor maps. These maps are instrumental in planning the surgical path to avoid critical areas, thereby reducing the risk of postoperative motor deficits and increasing the likelihood of achieving GTR (43).

These motor maps are exported and integrated into the neuronavigation systems, allowing real-time surgical guidance and adjustment for brain shift, enhancing the accuracy of the surgical approach (42). During surgery, the neuronavigation system, enriched with nTMS data, facilitates the use of direct cortical and subcortical stimulation to continuously monitor and preserve motor function. Additionally, preoperative PET imaging is fused with MRI and nTMS data within the neuronavigation system, providing critical metabolic information about the tumor and surrounding tissues, which helps in distinguishing healthy from diseased tissue (43).

### 4.8 Neuronavigation with augmented and virtual reality

**Figure 6:**
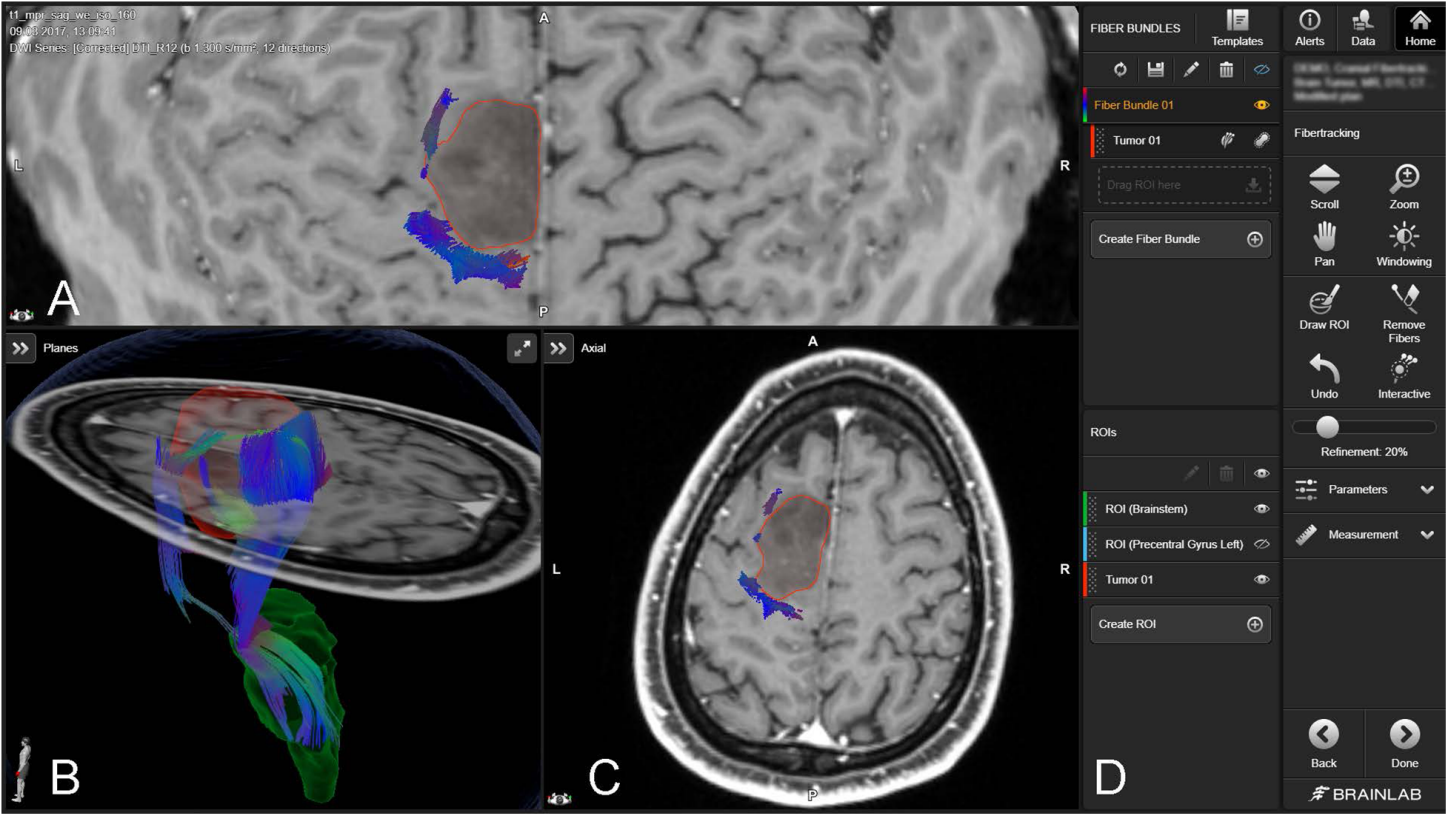
Fiber tracking with brainlab elements fibertracking (45). The figure includes a 3D rendering and cross-sectional MRI views [axial (**A,C**), and coronal (**B**)] of the brain, with a tumor outlined in red. The tractography data visualize the fiber bundles (depicted in various colors), indicating their spatial relationship to the tumor. The software interface on the right (**D**) provides functionalities for manipulating views, creating regions of interest (ROIs), and tracking fibers.

The integration of augmented reality (AR) and virtual reality (VR) with neuronavigation in glioma resection addresses the limitations of conventional techniques like electrical stimulation mapping, which provides limited spatial representation of vital white matter tracts such as the corticospinal tract (44). Advanced imaging modalities like DTI and high-definition fiber tractography (Figure 6) have become indispensable in noninvasive brain mapping, offering superior visualization of neural pathways, crucial for surgical planning and postoperative assessments (44). AR enhances surgical navigation by superimposing virtual images of brain fiber tracts onto real-world views, enabling surgeons to navigate complex structures more effectively, potentially increasing tumor resection rates, improving functional outcomes, and extending patient survival (44, 46).

The process of integrating AR and VR with neuronavigation begins with detailed MRI data acquisition, using both structural and diffusion-weighted imaging to capture comprehensive brain images. This data is then processed to classify and segment the tumor, incorporating DTI-based tractography to delineate essential neural tracts. The processed images are transferred to a neuronavigation platform which merges them with real-time surgical views in the AR system, creating an interactive surgical map (44, 46). Digital integration is established between this system and surgical instruments, especially the microscope, which is adapted to display AR images, overlaying 3D visualizations of the tumor and fiber tracts over the actual surgical area (44, 46). During surgery, the AR-guided strategy provides real-time visual guidance and updates, allowing surgeons to precisely navigate around critical structures, maximizing tumor resection while preserving vital brain functions (44). These technologies not only improve the EOR and reduce intraoperative complications but also enhance motor function outcomes and PFS (44, 46). Currently, AR and VR are only utilized on a small scale in the medical field.

### 4.9 Challenges in glioma surgery: tumor detection, neurological deficits, costs

One of the primary challenges is the inherent limitations in the ability of these systems to accurately visualize and detect tumor boundaries. For instance, the effectiveness of sodium fluorescein in fluorescence-guided surgery is contingent upon the disruption of the blood-brain barrier, which may not be uniformly disrupted across all areas of a glioma (41). This can lead to incomplete visualization of the tumor. Another challenge is the limitation of iMRI, particularly during awake craniotomy procedures (27). The patient's consciousness and potential movements, coupled with the extended duration of operations involving iMRI, can complicate the surgical process and limit the utility of this advanced imaging technique. Furthermore, issues with MRS signal quality, especially near convex or ventricular systems, can lead to inaccuracies in tumor delineation due to false-positive readings, posing a risk of either incomplete resection or unnecessary removal of healthy tissue (26). Similarly, the use of 2-D B-mode intraoperative ultrasound, without incorporating advanced ultrasound techniques, may limit the detection of smaller residual tumor volumes (28). Furthermore, the efficacy AR-intraoperative fiber tractography (AR-iFT), is constrained by the limitations of DTI techniques, such as parallax error and fiber tract crowding, which can affect the accuracy of surgical navigation (44). The specificity and sensitivity of these imaging modalities are critical for the precise identification and resection of tumor tissue.

The integration of neuronavigation and intraoperative imaging modalities aims to minimize neurological deficits by providing real-time guidance during surgery. However, the occurrence of transient deficits, and in some cases, new permanent neurological deficits, remains a significant concern. For example, the use of nTMS and IOM has been associated with a higher rate of transient deficits compared to IOM alone, although most of these deficits tend to resolve shortly after surgery (42). The risk of causing mechanical damage to motor eloquent tissue during surgery underscores the need for careful planning and execution, as well as the potential limitations of current technologies to fully mitigate these risks (42).

The implementation of neuronavigation systems and integrated imaging modalities comes with its own set of challenges, including the cost of the equipment, the need for specialized training for surgical teams, and the potential for prolonged operative times (24, 25, 31). The high cost of implementing and maintaining state-of-the-art neuronavigation and imaging systems, such as iMRI and 5-ALA fluorescence, places a significant financial burden on healthcare facilities, potentially limiting access to only well-resourced centers (31). Additionally one of the included studies found that the specificity of techniques to certain neuronavigation systems can constrain the choice of tools available to surgeons (26). For example, the necessity of using the BrainLab system for certain imaging modalities like 3D-MRS can limit interoperability with other neuronavigation systems (26). Another study questioned the cost-effectiveness of such advanced technologies, especially when the benefits in terms of extent of resection, clinical performance, or survival are not significantly superior to conventional methods (21). The requirement for special post-operative care, such as management of photosensitivity, and the complexity added to surgical procedures due to the need for continuous updating of the operative plan during surgery, further complicate their use (25, 32).

## 5 Limitations

The search methodology employed in this systematic review did not encompass the integration of virtual and augmented reality technologies in the neuronavigation processes for glioma resection. Recognizing the significance of these emerging technologies in enhancing surgical precision, we have broadened our discussion to provide a deeper understanding of their potential role in neuronavigation.

This systematic review did not include a comparative assessment of various neuronavigation techniques. Evaluating these modalities against each other could provide valuable insights to guide clinical decision-making and technology adoption in neurosurgical practices.

Some reviewed studies indicate lack of double-blinding, which is crucial to minimize bias in outcome assessment (28, 37). This concern was mitigated by having an independent blinded neuroradiologist assess the primary outcome. Another study faced challenges with non-prospective application of inclusion criteria and variability in surgical protocols (29). Furthermore, a common thread in several studies, is the issue of selection bias and the use of retrospective data, particularly when drawing from a single neurosurgical center or using retrospective control groups, thus limiting the generalizability and reliability of their findings (31, 34).

Additionally, the small sample size in many studies undermines the statistical power and generalizability of the results. The need for larger patient cohorts to detect subtle differences in outcomes and for larger, multicenter randomized controlled trials to validate findings and minimize biases is consistently emphasized (21, 24, 36, 38). The absence of long-term follow-up data restricts the understanding of the full impact of the surgical interventions on patient outcomes over time (25). These limitations collectively highlight the necessity for well-designed studies with larger, diverse patient populations, and standardized methodologies to ensure the reliability and applicability of research findings in the field of neurosurgery and glioma treatment.

## 6 Conclusions

This systematic review examines the application and effectiveness of neuronavigation systems and integrated imaging in glioma resection. The included studies highlight the significance of these technologies in improving patient safety and surgical outcomes by enhancing tumor visualization and differentiation, enabling intraoperative real-time adjustments, and preserving critical brain functions (22, 24, 25, 28, 37, 41, 43). These technologies also enhance patient safety and functional outcomes by facilitating surgery with real-time adjustments, resulting in improved quality of life and survival rates for patients. The primary challenge in neurosurgery imaging and navigation is the limitations of current technologies, such as iMRI and fluorescence-guided surgery, in accurately detecting tumor boundaries and the risks of neurological deficits, coupled with the high costs, and need for specialized training, which may limit their accessibility and effectiveness (24, 25, 27, 31, 41, 42). To fully realize the benefits and mitigate the limitations highlighted in the systematic review, further research is imperative to refine neuronavigation systems and integrated imaging technologies, focusing on enhancing accuracy in tumor boundary detection, reducing costs, and developing comprehensive training programs, thereby maximizing their potential in improving glioma resection outcomes. Future research should address these aspects, evaluating the financial implications and accessibility of these advanced technologies, and ensuring equitable access for patients. Ethical considerations, including informed consent and the potential risks vs. benefits of these tools, should also be explored to provide a comprehensive understanding of their impact on glioma surgery.

## Data Availability

The original contributions presented in the study are included in the article/Supplementary Material, further inquiries can be directed to the corresponding author.
