# Supplementary material for: Neuronavigation in glioma resection: current applications, challenges, and clinical outcomes
Source: Front Surg. 2024 Aug 6;11:1430567. doi: 10.3389/fsurg.2024.1430567 (PMC11334078; doi:10.3389/fsurg.2024.1430567)
Supplement: Supplementary file 1 [file Table1.docx]

Supplementary Material

Neuronavigation in Glioma Resection: Current Applications, Challenges, and Clinical Outcomes

Albert Joseph Sulangi, BS^1*^, Adam Husain, BS^2^, Haoyi Lei, BS^3^, Jessica Okun, DO, MS, FACOS^4^

^1^Nova Southeastern University, Dr. Kiran C. Patel College of Osteopathic Medicine – Tampa Bay Regional Campus, Clearwater, FL, USA

^2^University of Texas Medical Branch, Galveston, TX, USA

^3^Elson S. Floyd College of Medicine, Spokane, WA, USA

^4^Neurological Surgeon, Steward Medical Group, Fort Lauderdale, FL, USA

*** Correspondence:**Corresponding Author
as5106@mynsu.nova.edu

# Supplementary Data: Search Strategy

Search conducted on November 30, 2023

**MEDLINE (PubMed)**

Search: (neuronavigation OR brain mapping OR pointer-based navigation OR stealth OR microscope-based navigation OR multiple coordinate manipulator OR standard STP OR intraoperative MRI OR high-field MRI OR DTI-based neuronavigation OR image-guided resection OR fluorescence-guided resection OR 5-aminolevulinic acid OR image processing OR computer-assisted OR neuronavigation methods) AND (glioma OR glioblastoma OR eloquent area glioma)

Filters: Free full text, Clinical Trial, Randomized Controlled Trial, English, from 2012 - 2023

**Embase**

(‘neuronavigation’/exp OR neuronavigation OR ‘brain mapping’/exp OR ‘brain mapping’ OR ((‘brain’/exp OR brain) AND (‘mapping’/exp OR mapping)) OR ‘pointer-based navigation’ OR (‘pointer based’ AND (‘navigation’/exp OR navigation)) OR ‘stealth’/exp OR stealth OR ‘microscope-based navigation’ OR (‘microscope based’ AND (‘navigation’/exp OR navigation)) OR ‘multiple coordinate manipulator’ OR (multiple AND coordinate AND (‘manipulator’/exp OR manipulator)) OR ‘standard stp’ OR ((‘standard’/exp OR standard) AND (‘stp’/exp OR stp)) OR ‘intraoperative mri’/exp OR ‘intraoperative mri’ OR (intraoperative AND (‘mri’/exp OR mri)) OR ‘high-field mri’ OR (‘high field’ AND (‘mri’/exp OR mri)) OR ‘dti-based neuronavigation’ OR (‘dti based’ AND (‘neuronavigation’/exp OR neuronavigation)) OR ‘image-guided resection’ OR (‘image guided’ AND (‘resection’/exp OR resection)) OR ‘fluorescence-guided resection’ OR (‘fluorescence guided’ AND (‘resection’/exp OR resection)) OR ‘5-aminolevulinic acid’/exp OR ‘5-aminolevulinic acid’ OR (‘5 aminolevulinic’ AND (‘acid’/exp OR acid)) OR ‘image processing’/exp OR ‘image processing’ OR ((‘image’/exp OR image) AND (‘processing’/exp OR processing)) OR ‘computer assisted’ OR ‘neuronavigation methods’ OR ((‘neuronavigation’/exp OR neuronavigation) AND (‘methods’/exp OR methods))) AND (‘glioma’/exp OR glioma OR ‘glioblastoma’/exp OR glioblastoma OR ‘eloquent area glioma’ OR (eloquent AND (‘area’/exp OR area) AND (‘glioma’/exp OR glioma))) AND ([controlled clinical trial]/lim OR [randomized controlled trial]/lim) AND [2012-2023]/py AND ‘article’/it

**Web of Science**

(((TI=(neuronavigation OR brain mapping OR pointer-based navigation OR stealth OR microscope-based navigation OR multiple coordinate manipulator OR standard STP OR intraoperative MRI OR high-field MRI OR DTI-based neuronavigation OR image-guided resection OR fluorescence-guided resection OR 5-aminolevulinic acid OR image processing OR computer-assisted OR neuronavigation methods)) AND TI=(glioma OR glioblastoma OR eloquent area glioma)) AND AB=(neuronavigation OR brain mapping OR pointer-based navigation OR stealth OR microscope-based navigation OR multiple coordinate manipulator OR standard STP OR intraoperative MRI OR high-field MRI OR DTI-based neuronavigation OR image-guided resection OR fluorescence-guided resection OR 5-aminolevulinic acid OR image processing OR computer-assisted OR neuronavigation methods)) AND AB=(glioma OR glioblastoma OR eloquent area glioma) and Open Access and 2022 or 2021 or 2023 or 2020 or 2019 or 2018 or 2017 or 2016 or 2015 or 2014 or 2013 or 2012 (Publication Years) and Article (Document Types) and English (Languages)

# Supplementary Tables

## Keywords Identification

| **Source** | **Text words** | **MeSH Terms** |
| --- | --- | --- |
| Ganslandt O, Behari S, Gralla J, Fahlbusch R, Nimsky C. Neuronavigation: concept, techniques and applications. *Neurol India*. 2002;50(3):244-255. | pointer-based navigation  microscope-based navigation  iMRI  brain mapping | glioma  neuronavigation |
| Leroy HA, Delmaire C, Le Rhun E, Drumez E, Lejeune JP, Reyns N. High-field intraoperative MRI and glioma surgery: results after the first 100 consecutive patients. *Acta Neurochir (Wien)*. 2019;161(7):1467-1474. doi:10.1007/s00701-019-03920-6 | high-field MRI  iMRI  glioma | Karnofsky Performance Status  neuronavigation  glioma |
| Akay A, Nasirov R, Ruksen M, Eraslan C, Islekel S. DTI-based neuronavigation guided eloquent area glioma resection with awake craniotomy: supra-functional resection of eloquent area gliomas. *Turk Neurosurg*. 2019;29(5):759-767. doi:10.5137/1019-5149.JTN.25779-19.1 | DTI-based neuronavigation  eloquent area glioma  glioblastoma  mapping |  |
| Panciani PP, Fontanella M, Schatlo B, et al. Fluorescence and image guided resection in high grade glioma. *Clin Neurol Neurosurg*. 2012;114(1):37-41. doi:10.1016/j.clineuro.2011.09.001 | image guided resection  fluorescence guided resection  5-aminolevulinc acid | image-processing, computer-assisted  neuronavigation methods |

## Full-Text Review for Eligibility Assessment

|  | **Source, country** | **Study design, number of participants** | **Patient characteristics** | **Aim** | **Results** | **Conclusion** | **Eligibility** |
| --- | --- | --- | --- | --- | --- | --- | --- |
| 1 | Wang et al,^43^ 2023, China | Prospective randomized controlled trial, n = 120 | The study included individuals aged 18 to 75, of any gender, with high-grade gliomas eligible for surgery, newly diagnosed and not having received specific oncologic treatment, and possessing an electrocorticography (ECoG) score of ≤2. | To evaluate the clinical effectiveness of sodium fluorescein-guided microsurgery in patients with high-grade gliomas by comparing its efficacy with neuronavigation microsurgery alone, employed as the CG, and neuronavigation microsurgery combined with sodium fluorescein-guided microsurgery, utilized in the SG. | The study group achieved a significantly higher Gross Total Resection Rate (GTRR) with a shorter operation time, comparable intraoperative bleeding and hospital stay, but experienced a significant decline in Karnofsky Performance Score (KPS) and National Institutes of Health Stroke Scale (NIHSS) scores post-treatment; adverse effects were similar. While the SG showed significantly higher overall survival (OS), there was no significant difference in progression-free survival (PFS) compared to the CG. | Fluorescein-guided microsurgery can dramatically improve total resection rate, postoperative neurological functional status, and overall survival with higher efficacy and safety in patients with high-grade gliomas. | Include |
| 2 | Near-infrared window II fluorescence image-guided surgery of high-grade gliomas prolongs the progression-free survival of patients. Shi X, Zhang Z, Zhang Z, et al. 2022, China | Randomized controlled trial, n = 40 | Enrolled were patients aged 18-75 with suspected newly diagnosed, untreated gliomas eligible for surgical resection, having a Karnofsky Performance Score (KPS) over 70, presenting with a single solid contrast-enhanced lesion not in the midline, basal ganglia, cerebellum, or brain stem, possessing normal heart, bone marrow, liver, and kidney function, compatible with follow-up, negative pregnancy test for women of child-bearing age, agreement from both male and female patients to use effective contraception, voluntary signing of the informed consent form, and possessing complete medical records for analysis. | To evaluate medical value and treatment efficacy ofNIR-II image-guided surgery | In the study, enrollment included 15 glioblastoma (GBM) and 4 WHO grade III glioma patients in the Fluorescence-Guided Surgery (FGS) group, and 18 GBM and 4 WHO grade III glioma patients in the White Light Surgery (WLS) group, with a 100% detection rate of NIR-II fluorescence for GBM in the FGS group; FGS demonstrated a significantly increased complete resection rate for GBM compared to WLS, and the FGS group exhibited significantly prolonged progression-free survival (PFS) and overall survival (OS) with no recurrence observed in WHO grade III glioma patients. | Near-infrared window II fluorescence-guided surgery (NIR-II FGS) significantly improves the complete resection rate of glioblastoma (GBM) compared to conventional white light surgery (WLS), resulting in substantially enhanced survival for GBM patients. | Exclude. Off-topic focus. Limited neuronavigation utilization. |
| 3 | Incekara et al,^21^ 2021, The Netherlands | Randomized controlled trial, n = 50 | Patients 18 years or older with a newly diagnosed, contrast-enhancing presumed glioblastoma, deemed totally respectable, presenting at the Erasmus MC (Rotterdam, The Netherlands) were enrolled and randomized (1:1) into intraoperative B-mode ultrasound guided surgery or resection under standard neuronavigation. | To assess the value of intraoperative B-mode ultrasound guided surgery on the extent of glioblastoma resection | Eight (35%) of 23 patients in the intraoperative B-mode ultrasound group and two (8%) of 24 patients in the standard surgery group underwent complete resection (p = 0.036). Baseline characteristics, neurological outcome, functional performance, quality of life, complication rates, overall survival and progression-free survival did not differ between treatment groups (p > 0.05). | Intraoperative B-mode ultrasound enables complete resection more often than standard surgery without harming patients and can be considered to maximize the extent of glioblastoma resection during surgery. | Include |
| 4 | First-in-human study of PET and optical dual-modality image-guided surgery in glioblastoma using 68Ga-IRDye800CW-BBN. Li D, Zhang J, Chi C, et al. 2018, China | Prospective Cohort Study, n = 14 | In this cohort of 14 patients (12 males, 2 females; median age 47), 8 had newly diagnosed and 6 had recurrent glioblastoma multiforme (GBM), with 2 of the recurrent cases initially diagnosed as anaplastic astrocytoma. According to Sawaya and colleagues’ classification, 10 patients had grade III lesions, 2 had grade II, and 2 had grade I lesions, with a median preoperative tumor volume of 55.49 cm3 based on contrast-enhanced T1W MRI. | To explore the feasibility of the dual-modality imaging tracer and intraoperative NIRF navigation system for the surgical resection of gliomas while avoiding severe neurological deficits | The near-infrared intraoperative system allowed precise resection of orthotopic tumors in mice, and in a translational cohort of 14 GBM patients, there was a strong correlation between preoperative positive PET uptake and intraoperative NIRF signal. Tumor fluorescence signals were significantly higher than those from adjacent brain tissue, and fluorescence-guided sampling demonstrated high sensitivity (93.9%) and specificity (100%) compared to pathology, with a safe tracer and satisfactory resection outcomes, resulting in an 80% progression-free survival at 6 months for the cohort. | The initial study shows that the novel dual-modality imaging technique is feasible for integrated pre- and intraoperative targeted imaging, utilizing the same molecular receptor, leading to enhanced intraoperative visualization of glioblastoma multiforme (GBM) and facilitating maximum safe resection. | Exclude. Off-topic focus. Limited neuronavigation utilization. |
| 5 | Accuracy of VarioGuide frameless stereotactic system against frame-based stereotaxy: prospective, randomized, single-center study. Bradac O, Steklacova A, Nebrenska K, Vrana J, de Lacy P, Benes V. 2017, United Kingdom | Prospective, randomized, single-center study, n = 53 | Patient’s age >18 years, brain pathology indicating brain biopsy, patient willingness, and ability to sign informed consent for participation in the study. | To determine VarioGuide’s accuracy and diagnostic yield and to compare this with  frame-based (FB) stereotaxy. | The study found no significant differences between the Freehand biopsy (FB) and Virtual Navigation-guided biopsy (VG) groups in terms of lesion volume, target distance, angle deviation, diagnostic yield, and operating time; however, overall patient discomfort was significantly higher in the FB group, with a visual analog scale score of 2.5 compared to 1.2 in the VG group. | The Virtual Navigation-guided (VG) system demonstrated comparable trajectory accuracy, complication rates, and diagnostic yield to the traditional Freehand Biopsy (FB) stereotaxy, considered the “gold standard” for brain biopsy, while being better accepted by patients. | Exclude. Off-topic focus. Limited neuronavigation utilization. |
| 6 | Picht et al,^29^ 2015, USA | Controlled observational study, n = 93 | Patients who had glioblastoma located within, or in the immediate vicinity of, the primary motor cortex and/or the pyramidal tract. | To isolate the impact of navigated transcranial magnetic stimulation (nTMS) on surgical outcome in glioblastoma treatment | The addition of preoperative nTMS mapping data to a clinical routine already incorporating preoperative fiber tractography and intraoperative neuronavigation and electrophysiology was shown to improve surgical outcomes by increasing the extent of resection, without compromising patient safety or long-term functional outcomes in comparison to the concurrent non-TMS CG. | The results of this extensive controlled observational study provide robust evidence that augmenting standard intraoperative cortical mapping with preoperative navigated transcranial magnetic stimulation (nTMS) motor mapping and nTMS-based fiber tracking enhances surgical outcomes without compromising functional results in glioblastoma patients within motor eloquent areas. Furthermore, preoperative nTMS motor mapping has the potential to broaden the pool of patients eligible for safe surgical treatment, and the study’s pragmatic design suggests a high level of external validity. | Include |
| 7 | Microrecording and image-guided stereotactic biopsy of deep-seated brain tumors. Iijima K, Hirato M, Miyagishima T, et al. 2015, Japan | Retrospective observational study, n = 12 | Patients with small deep-seated brain tumor, in which image-guided stereotactic biopsy was performed with the aid of depth microrecording | To evaluate the effectiveness of image-guided stereotactic biopsy in combination with depth microrecording for obtaining samples of small deep-seated brain tumors | The study found that the diagnostic yield for image-guided stereotactic brain tumor biopsy, particularly with depth microrecording, was high at 100%, comparable to 93.8% in the group without microrecording. The postoperative complication rate was low, and there were no mortalities despite dealing with small and deep-seated target lesions. Depth microrecording revealed patterns of neural activity changes along the trajectory, and close correlations were observed between electrophysiological, MRI, and histological findings in some cases. | Image-guided stereotactic biopsy with depth microrecording is a safe procedure that offers real-time accurate positional information, distinguishes tumors from brain structures during surgery, and holds potential for investigating the epileptogenicity of brain tumors. | Exclude. Off-topic focus. Study not specific to glioma. |
| 8 | Coburger et al,^24^ 2015, Germany | Prospective cohort study with retrospective matched pair assessment, n = 177 | The sample characteristics include two cohorts: the 5-ALA&iMRI cohort, prospectively including patients with contrast-enhancing lesions eligible for gross total resection (GTR) and a final histopathological diagnosis of GBM WHO°IV from July 2012 to February 2014, and the control cohort (iMRI) based on a retrospective assessment of all patients who had iMRI-assisted surgery from September 2008 to July 2012. Inclusion criteria for both cohorts involve intended GTR, complete follow-up, and assessment of MGMT promoter methylation status, with matching criteria including tumor volume, recurrent surgery, eloquent location, and age. | To assess impact of additional use of 5-ALA in iMRI-assisted surgery of GBMs on extent of resection (EoR), progression free survival (PFS) and overall survival (OS). | The 5-ALA&iMRI group achieved a higher gross total resection rate (100%) and mean extent of resection (99.7%) compared to the iMRI-alone group (82% GTR, 97.4% EoR), with similar complication rates (21% iMRI, 27% 5-ALA&iMRI), nPND (6% in both groups), and no significant differences in median progression-free survival (PFS) and overall survival (OS). | The study revealed a significant increase in the extent of resection (EoR) when combining 5-ALA&iMRI compared to using iMRI alone, and maximizing EoR did not result in an increase in complications or neurological deficits when neurophysiological monitoring was employed in eloquent lesions. However, the study did not provide a conclusive determination on whether a further increase in EoR translates to improved patient progression-free survival and overall survival. | Include |
| 9 | Zhang et al,^33^ 2015, China | Prospective cohort study, n = 217 | Right-handed patients with single, supratentorial, primary gliomas in the dominant hemisphere with a distance less than 20 mm from the tumor to language-associated structures. | To evaluate the impact of intraoperative magnetic resonance imaging (iMRI) and functional neuronavigation on surgical outcome in patients with gliomas involving language areas | Among 198 glioma patients, the SG (112 patients) demonstrated significantly higher rates of extensive resection and radiological gross total resection, better language functions at 6-month follow-up, a much lower occurrence of postoperative new aphasia, and dramatically prolonged progression-free and overall survival for glioblastoma patients compared to the CG (86 patients). | The use of iMRI and functional neuronavigation could enhance tumor resection, reduce language deficits in patients with gliomas affecting language areas, and potentially extend survival time for those with glioblastomas. | Include |
| 10 | Margetis et al,^34^ 2015, USA | Prospective Phase I–II study, n = 10 | Patients with high- and low-grade gliomas | To investigate the safety and efficacy of a technique for marking the deep margins of intraaxial tumors with stereotactic injection of Indigo Carmine dye. | In a study involving 17 injections in 10 enrolled glioma patients, the intraoperatively identified injection points were successfully resected, exhibiting a reproducible staining pattern with a vividly colored and demarcated sphere of stained tissue approximately 5 mm in diameter; postoperative MRI verified the resection of all injection points, resulting in a mean extent of tumor resection of 97.1%, although one patient developed a brain abscess on postoperative Day 16 requiring additional surgical treatment. | Stereotactic injection of Indigo Carmine dye is a viable method for demarcating multiple deep tumor margins, easily identified intraoperatively with standard white light microscopy, suggesting its potential to improve the accuracy of frameless stereotactic navigation and increase the extent of resection for intraaxial tumors. | Include |
| 11 | Kubben et al,^32^ 2014, Belgium | Randomized controlled trial, n = 14 | Patients with supratentorial brain tumor-suspected to be glioblastoma on contrast‑enhanced diagnostic MRI, indication for gross total resection (GTR) of the tumor, age 18 years or older, WHO Performance Scale (WPS) 2 or better, ASA class 3 or better, adequate knowledge of the Dutch or French language, and informed consent. | To assess whether iMRI‑guided surgery leads to increased EOTR compared with cNN‑guided surgery, and whether health‑related quality of life (HRQOL) differs between these two approaches | There was no statistically significant difference in median residual tumor volume or survival between the conventional neuronavigation (cNN) and iMRI groups, and clinical performance and health-related quality of life outcomes were not significantly different, with descriptive statistics applied due to a limited sample size. | The interim analysis of a randomized trial comparing iMRI-guided glioblastoma resection with cNN-guided resection indicates no advantage in terms of extent of resection, clinical performance, and survival for the iMRI group. | Include |
| 12 | Fluorescence and image guided resection in high grade glioma. Panciani PP, Fontanella M, Schatlo B, et al. 2011, Italy | Prospective cohort study, n = 54 | Patients with suspected, newly diagnosed, untreated malignant  gliomas and who were amenable to complete resection of contrast-enhancing tumor. | To analyze the advantages and limitations of fluorescence and image-guided resection in high-grade glioma, with a multicentric prospective design to evaluate the accuracy of each method, including the role of 5-aminolevulinic acid and neuronavigation | The study noted an enhancement in the sensitivity of 5-aminolevulinic acid (5-ALA) through combined use with neuronavigation, albeit at the cost of a significant reduction in specificity, emphasizing the need for critical surgeon skills in the judicious application of auxiliary techniques, and advocating for a large-scale study to enhance the assessment of multimodal approaches. | Fluorescence- and image-guides, particularly 5-aminolevulinic acid (5-ALA), can effectively detect pathological tissue not visible through conventional surgical strategies, and while neuronavigation can enhance the sensitivity of 5-ALA, its combined use may reduce specificity, emphasizing the importance of guiding surgery based on surgical anatomy, supplemented by auxiliary techniques. | Exclude. Limited data on post-operative results. |
| 13 | Akay et al,^38^ 2019, Turkey | Retrospective cohort study, n = 18 | Patients with a diagnosis of glial tumour located in, or close to, the eloquent area. | To provide an overview of their awake craniotomy practices utilizing DTI-based neuronavigation (DTI-bN) and cortical and subcortical stimulation (CSC) for glioma resection in eloquent areas, with a focus on revealing the clinical outcomes in terms of neurological morbidity and residual tumor volume. | In a cohort of 18 patients undergoing awake craniotomy for glioma resection in eloquent areas, 50% achieved gross total resection, 38.8% underwent near total resection, and 11.1% had a subtotal resection, with intraoperative neurological deterioration observed in 14 patients, leading to permanent deficits in two patients as per sixth-month postoperative neurological examinations. | In this study, the concept of ‘supra-functional resection’ is introduced to describe the maximal extent of glioma resection that avoids permanent neurological deficits, utilizing the combined approach of DTI-based neuronavigation (DTI-bN) and cortical-subcortical electrical stimulation to preserve functional areas during eloquent area glioma resection and minimize damage to white matter fiber tracts and cortical structures due to brain shift. | Include |
| 14 | Segmentation-based registration of ultrasound volumes for glioma resection in image-guided neurosurgery. Canalini L, Klein J, Miller D, Kikinis R. 2019, Norway | Methodological study, n = 17 | Clinical cases of low-grade gliomas (Grade II) acquired on adult patients between 2011 and 2016 at St. Olavs University Hospital, Norway | To address the issue of brain shift during glioma removal surgeries by developing and evaluating a fully automated segmentation-based registration method for ultrasound (US) volumes acquired at different stages of neurosurgery to provide an updated and reliable view of the resection cavity. | The proposed method significantly decreases the mean target registration error (mTRE) between volumes obtained before the opening of the dura mater and during resection, enhancing the accuracy of spatial mapping in neurosurgical procedures. | The segmented structures prove to be effective for registering ultrasound volumes at various neurosurgical phases, offering a solution to mitigate brain shift in procedures using intraoperative ultrasound data. | Exclude. Limited data on pre- and post-operative results. |
| 15 | c(RGDyk)-modified nanoparticles encapsulating quantum dots as a stable fluorescence probe for imaging-guided surgical resection of glioma under the auxiliary UTMD. Wu QL, Xu HL, Xiong C, et al. 2020, China | Randomized controlled trial, n = 42 | Adult male Sprague-Dawley (SD) rats (200–280 g) injected with C6 glioma cells | To analyze novel targeting material (c(RGDyk)-poloxamer-188) and its application in constructing glioma-targeted quantum dots (QDs)-encapsulated polymer nanoparticles (NPs) for imaging-guided surgical resection of glioblastoma. | The study introduced a novel glioma-targeted nanoparticle, QDs-c(RGDyk)NP, synthesized for imaging-guided surgical resection of glioblastoma (GBM), demonstrating specific targeting to glioma tissue and accurate surgical resection when combined with ultrasound-targeted microbubble destruction (UTMD). | The results suggest that QDs-c(RGDyk)NP holds promise as a potential imaging probe for improving the precision of GBM surgery. | Exclude. Off-topic focus. Limited neuronavigation utilization. |
| 16 | Indications of 5-aminolevulinic acid and intraoperative MRI in glioma surgery: first cases in Latin America in a single reference center. Ramina R, da Silva EB, Constanzo F, Neto MC. 2018, Brazil | Retrospective observational study, n = 62 | 62 patients, comprising 43 males and 21 females with a mean age of 51.25 years, underwent a total of 64 surgeries for intracranial glioma, and all patients had a preoperative Karnofsky Performance Status (KPS) greater than 70%. | To assess the combined use of 5-aminolevulinic acid (5-ALA) and intraoperative magnetic resonance imaging (iMRI) in glioma surgery, evaluating the extent of resection (EOR) and providing insights into the effectiveness of these techniques, particularly in cases with varying 5-ALA fluorescence and gliomas infiltrating eloquent areas | In cases where tumors exhibited intraoperative 5-ALA fluorescence, 28 out of 46 achieved a safe 5-ALA free resection. For tumors lacking 5-ALA fluorescence, iMRI findings guided the extent of resection, leading to complete resection in 11 cases, while complete resection was avoided in gliomas infiltrating eloquent areas. | The combined use of 5-ALA and iMRI in glioma surgery improved outcomes, ensuring the safest maximal extent of resection (EOR). In 5-ALA positive cases, fluorescence was particularly beneficial, while in 5-ALA negative cases, iMRI played a decisive role in guiding EOR. | Exclude. Limited Postop results |
| 17 | Zhang et al,^31^ 2016, China | Prospective study, n = 15 | Patients who had a diagnosis of cerebral glioma were eligible to participate in this study | To integrate 3D 1H-MRS into neuronavigation and assess the feasibility and validity of metabolically based glioma resection | This revealed statistically significant differences in metabolic volumes based on Cho-NAA index (CNI) thresholds, particularly in low-grade glioma (LGG) and high-grade glioma (HGG) groups. Despite these differences, all patients demonstrated no tumor progression in the 1-year follow-up. | The study successfully integrated three-dimensional MRS maps and intraoperative navigation, applying optimal Cho-NAA index thresholds for both low-grade and high-grade gliomas, demonstrating improved outcomes for glioma resection through the integration of 3D 1H-MRS with structural imaging. | Include |
| 18 | Chen et al,^35^ 2017, China | Retrospective cohort study, n = 42 | Over a 4-year period between July 2010 and July 2014, 51 consecutive patients with histologically verified insular primary glioblastoma (GBM, glioma grade IV) or anaplastic astrocytoma (grade III) underwent treatment using combined high-field intraoperative magnetic resonance imaging (iMRI) and functional neuronavigation, while 22 patients with insular primary GBM or anaplastic astrocytoma underwent conventional neuronavigation. | To evaluate the impact of high-field iMRI and functional neuronavigation on the surgical resection of insular HGG. | In patients undergoing iMRI-assisted surgery for insular high-grade glioma, the detection and resection of residual tumors significantly increased the median extent of resection, resulting in a smaller mean residual tumor volume, improved Karnofsky Performance Score, and better progression-free and overall survival compared to cNN surgery. | The combination of high-field intraoperative magnetic resonance imaging (iMRI) and functional neuronavigation enhances the extent of resection and reduces morbidity in insular high-grade glioma surgery, with aggressive resection correlating with improved overall and progression-free survival. | Include |
| 19 | 5-aminolevulinic acid fluorescence-guided resection of 18F-FET-PET positive tumor beyond gadolinium enhancing tumor improves survival in glioblastoma. Müther M, Koch R, Weckesser M, et al. 2019, Germany | Prospective observational study, n = 31 | adult patients with Gd+ GBM | To investigate Gd+and 18F-FET-PET tumor volumes after fluorescence-guided resection in GBM patients and to correlate residual volumes with survival data | Out of 31 patients, 18 with residual 5-ALA-derived fluorescent tissue, and a median 18F-FET-PET volume of 17.82 cm3, showed incomplete resection of gadolinium-enhancing tumor, while patients without residual fluorescence (median 18F-FET-PET volume 1.20 cm3) achieved complete resection, and an 18F-FET-PET volume above 4.3 cm3 was associated with worse overall survival, even in patients with no residual contrast-enhancing tumor on MRI. | Postoperative 18F-FET-PET volumes predict OS and PFS. Resection of 5-ALA-derived fluorescence beyond gadolinium enhancing tumor tissue leads to lower postoperative 18F-FET-PET tumor volumes and improved OS and PFS without additional deficits. | Exclude. Off-topic focus. Limited neuronavigation utilization. |
| 20 | Chan et al,^39^ 2018, Hong Kong, SAR | Retrospective study, n = 16 | Patients with known history of malignant glioma (recurrence or second resection of newly diagnosed malignant glioma); specific MRI features suggesting malignant glioma (heterogenous gadolinium contrast enhancement with or without central necrosis). | To evaluate the utility of 5-Aminolevulinic Acid (5-ALA) in guiding glioma resection, with a focus on achieving total removal of the tumor and assessing the impact of the time window for 5-ALA administration on the extent of resection. | In the study, 5-ALA was used in patients with confirmed or presumed malignant glioma, resulting in positive fluorescence in 15 cases with varying intensity; the correlation between extent of resection and the duration from 5-ALA ingestion to microscope visualization was significant, and two patients experienced temporary visual field defects, while one developed hemiparesis post-surgery. | 5-ALA is a beneficial intraoperative guidance tool for tumor resection, enhancing the likelihood of achieving total removal; however, its optimal use is recommended within a specific window period of 4 to 12 hours. | Include |
| 21 | Bettag et al,^28^ 2021, Germany | Retrospective study, n = 12 | Patients with primary GBM presumed to be noneloquently located, only those patients with data sets containing 3D T1- weighted MR images before and after administration of a contrast agent (0.1 mmol/ml gadobutrol per kg body weight, Gadovist, Bayer Vital GmbH) pre- and post-tumor resection were included. | To determine via volumetric analyses of the EOR whether endoscope-assisted FG resection enables supratotal resection beyond the borders of contrast enhancement | In GBM patients undergoing endoscopic surgery after complete microscopic FG resection, residual fluorescent tissue undetected with the microscope was histopathologically confirmed, resulting in a mean postoperative volume of nonenhancing but fluorescent tumor tissue at 62.0 cm3 and a relative resected volume of 244.7% compared to the contrast-enhancing tumor volume (p < 0.001). | The combined microscopic and endoscopic FG resection of GBM significantly enhances the extent of resection (EOR) and enables the attainment of a supratotal resection beyond the borders of contrast enhancement in noneloquently located GBM. | Include |
| 22 | Fujii et al,^36^ 2022, Japan | Retrospective cohort study, n = 11 | Patients who underwent glioma surgery with intraoperative magnetic resonance imaging (iMRI) for total tumor resection between July 2018 and October 2020 | To assess the advantages and disadvantages of using low-field intraoperative magnetic resonance imaging (iMRI) compared to non-use of iMRI during glioma surgery | The iMRI group achieved a higher gross total resection rate (73%) compared to the CG (18%), with no significant difference in transient neurological deficits or unintended reoperations; mean operating time did not differ between the groups. | Low-field iMRI enhances gross total resection rates, minimizes unintended reoperations, and does not prolong operating time, indicating its potential to streamline decision-making and reduce procedural hesitation in glioma surgery. | Include |
| 23 | Hauser et al,^26^ 2016, Switzerland | Prospective clinical study, n = 14 | Patients operated on at the University Hospital Zurich, Switzerland, between August 2009 and June 2011 with a primary  diagnosis of glioblastoma (no other tumor entity, metastases, recurrent glioblastoma, or malignant transformation of a previously known lower-grade glioma). Patients were only enrolled if the T1 contrast-enhancing areas on preoperative MRI were considered completely respectable, taking into account the anatomic location of the tumor. | To evaluate 5-ALA fluorescence combined with subsequent low-field iMRI for resection control in glioblastoma surgery. | In 13 of 14 cases, the diagnosis was glioblastoma multiforme. One lymphoma and 1 case without fluorescence were excluded. In 11 of 12 operations, residual contrast enhancement on iMRI was found after complete resection of 5-ALA fluorescent tissue. In 1 case, the iMRI enhancement was in an eloquent area and did not undergo a biopsy. The 28 biopsies of areas suspicious for tumor on iMRI in the remaining 10 cases showed tumor in 39.3%, infiltration zone in 25%, reactive central nervous system tissue in 32.1%, and normal brain in 3.6%. | 5-ALA fluorescence–guided resection may leave some glioblastoma tissue undetected. MRI might detect areas suspicious for tumor even after complete resection of all fluorescent tissue; however, due to the limited accuracy of iMRI in predicting tumor remnant (64.3%), resection of this tissue has to be considered with caution in eloquent regions. | Include |
| 24 | Lu et al,^37^ 2018, China | Prospective cohort study, n = 40 | 40 patients with a glioma located in the eloquent brains areas who underwent treatment in the Neurosurgery Department of Peking University International Hospital between December 2015 and August 2017. The experimental group included 20 patients treated using iMRI assistance technology (iMRI group). The remaining 20 patients underwent treatment by conventional neuronavigation (non-iMRI group). | To assess the applicability of 3.0 T high-field intraoperative magnetic resonance imaging (iMRI) combined with multimodal neuronavigation in the resection of gliomas in eloquent brain areas | The study found that the use of 3.0 T high-field intraoperative magnetic resonance imaging (iMRI) combined with multimodal neuronavigation in the resection of gliomas in eloquent brain areas resulted in a higher resection rate, improved postoperative functional outcomes, and comparable infection rates, despite a longer operative time compared to cNN surgery. | The study demonstrates that the integration of intraoperative magnetic resonance imaging (iMRI) with multimodal neuronavigation enhances real-time navigation during glioma resection, leading to increased resection rates in eloquent brain regions, preservation of neurological functions, and a manageable infection rate, highlighting its potential as an advanced assisting technology in microneurosurgery with broad clinical applications. | Include |
| 25 | Fluorescence-guided high-grade glioma surgery more than four hours after 5-aminolevulinic acid administration. Maragkos GA, Schüpper AJ, Lakomkin N, et al. 2021, USA | Retrospective study, n = 16 | All patients receiving 5-ALA for resection of radiographic high-grade glioma (HGG) were screened at two separate institutions between 2017 and 2020. Patients were included if they received anesthesia induction more than 4 h following 5-ALA administration. Patients were excluded if they received anesthesia induction within 4 h of 5-ALA, or if they had non-HGG tissue upon histopathology. | To demonstrate that there is adequate intraoperative fluorescence in cases undergoing surgery more than 4 h after 5-ALA administration and compare survival and radiological recurrence to previous data. | Sixteen patients, predominantly involving eloquent cortex, underwent surgery with 5-ALA fluorescence, exhibiting adequate intraoperative fluorescence, residual postoperative contrast enhancement in the majority, and a mean progression-free survival of 5 months, with 37.5% mortality and associated functional and neurological assessments | The study shows that performing 5-ALA-guided high-grade glioma resection more than 4 hours after administration is safe, yielding clinical results comparable to previous reports, suggesting that relaxing timing restrictions could enhance workflow in neurosurgical centers without increasing patient risk. | Exclude. Off-topic focus. Limited neuronavigation utilization. |
| 26 | Schatlo et al,^27^ 2015, Switzerland | Retrospective study, n = 200 | Patients aged 18 years or older with high-grade glioma who underwent treatment from 2003 to 2011 | To compare progression-free and overall survival rates in patients with high-grade gliomas undergoing surgical resection with the combination of 5-aminolevulinic acid (5-ALA) and iMRI against a CG without iMRI | The combination of 5-aminolevulinic acid (5-ALA) and iMRI enhances the achievement of gross total resection, but it does not significantly impact overall or progression-free survival in patients with high-grade glioma when adjusted for resection status. | Complete removal of the tumor significantly influences survival in high-grade glioma patients, particularly when using surgical adjuncts like iMRI and 5-ALA. | Include |
| 27 | Contribution of combined intraoperative electrophysiological investigation with 3-T iMRI for awake cerebral glioma surgery: comprehensive review of the clinical implications and radiological outcomes. Ghinda D, Zhang N, Lu JF, Yao CJ, Yuan SW, Wu JS. 2016, China | Retrospective study, n = 160 | Patients who underwent an awake craniotomy and iMRI for resection of eloquent area glioma during the 53 months between January 2011 and June 2015 | To assess the clinical efficiency of combined awake craniotomy with 3-T iMRI–guided resection of gliomas adjacent to eloquent cortex performed at a single center; to explore the contribution of iMRI to surgeons’ learning process of maximal safe resection of gliomas. | During a 24.8-month follow-up, 46.2% experienced short-term neurological worsening and 8.7% long-term worsening; median and mean EOR were 100% and 92%, respectively, with complete radiographic resection in 60.4% of patients. Low-grade glioma had a significantly lower GTR rate (89.06% ± 19.6%) than high-grade glioma (96.4% ± 9.1%) (p = 0.026). Thirty patients (28.3%) had additional resection post-iMRI, resulting in a 10.1% EOR increase, and multivariate Cox modeling revealed that the final EOR significantly predicted PFS (HR 0.225, 95% CI 0.070–0.723, p = 0.012). For high-grade glioma, GTR (p = 0.033), short-term motor deficit (p = 0.027), and WHO grade (p = 0.005) independently influenced OS, while performing further resection after iMRI (p = 0.083) and achieving GTR (p = 0.05) demonstrated a PFS benefit trend for low-grade glioma. Over time, the rate of post-iMRI resection decreased by 26.1% (p = 0.005), with a nonsignificant decline in short-term (p = 0.101) and long-term (p = 0.132) neurological deficits. | Combined awake craniotomy and iMRI is a safe and efficient technique allowing maximal safe resection of eloquent area gliomas with possible subsequent OS and PFS benefits. Although there is a learning curve for applying this technique, it can also improve the surgeon’s ability in eloquent glioma surgery. | Exclude. Off-topic focus. Limited neuronavigation utilization. |
| 28 | De Witt Hamer et al,^22^ 2013, France and The Netherlands | Retrospective cohort study, n = 108 | Patients over 17 years with diffusely infiltrative glioma of WHO grade II or infiltrative glioma largely showing WHO grade II characteristics with an anaplastic focus of mitotic activity. | To quantitatively compare glioma resection outcomes between surgical teams with varying levels of experience in intraoperative brain stimulation mapping, utilizing a novel approach called resection probability maps (RPMs) to assess and standardize the quality of resective surgery. | Neurological outcomes were similar between teams, and the resection probability maps showed very little differential resection in both left and right-sided tumors. | Resection probability maps (RPMs) offer a standardized quantitative approach for assessing resective glioma surgery quality, avoiding brain location bias, and demonstrating the robustness of stimulation mapping, which, irrespective of surgical experience, ensures independent and favorable neurological and functional-based resection outcomes, supporting broader implementation. | Include |
| 29 | 5-aminolevulinic acid (5-ALA)-induced protoporphyrin IX fluorescence by glioma cells-a fluorescence microscopy clinical study. Pacioni S, D’Alessandris QG, Giannetti S, et al. 2022, Italy | Observational study, n = 27 | Patients undergoing fluorescence-guided tumor resection, assigned to receive 5-aminolevulinic acid (5-ALA) with Gliolan®, administered orally at a dosage of 20 mg/kg bodyweight, scheduled 3 hours before anesthesia induction, and pre-treated with 12 mg/day of dexamethasone for a minimum of 2 days before 5-ALA administration | To evaluate the efficacy of 5-aminolevulinic acid (5-ALA)-induced PpIX fluorescence in neurosurgery for identifying high-grade glioma tissue by assessing the actual number of fluorescence-positive tumor cells in both low-grade and high-grade gliomas and investigating the ability of 5-ALA to cross the blood–brain barrier | The study found that in high-grade gliomas, 32.7–75.5 percent of cells displayed 5-aminolevulinic acid (5-ALA) induced PpIX fluorescence, while in low-grade gliomas, tumor cells did not fluoresce following 5-ALA, and immunofluorescence suggested that 5-ALA does not cross the un-breached blood–brain barrier, emphasizing the limited usefulness of 5-ALA-induced PpIX fluorescence in surgery for low-grade glioma, particularly when the blood–brain barrier is preserved. | The study emphasizes the established role of 5-aminolevulinic acid (5-ALA)-induced PpIX fluorescence in high-grade glioma surgery but highlights its limited usefulness in low-grade glioma surgery, particularly when the blood–brain barrier is preserved. | Exclude. Limited data on pre- and post-operative results. |
| 30 | Hou et al,^23^ 2022, China | Retrospective study, n = 40 | The sample included 40 patients, with a gender distribution of 22 women and 18 men, and a mean age of 48.28 ± 12.66 years. The majority of tumors were high-grade gliomas (77.5%), and 62.5% were located in eloquent areas, with a mean tumor volume of 61.64 ± 44.53 cm3. | To evaluate the effectiveness of a full-course resection control strategy combining intraoperative ultrasound (iUS) and iMRI in the surgical treatment of supratentorial gliomas, assessing its impact on surgical outcomes, resection control, and efficiency compared to iMRI-assisted glioma surgery. | The extent of resection was high at 95.43%, with a gross total resection rate of 72.5%, and a median residual tumor size of 6.39 cm3; 5% of patients experienced permanent neurological deficits, 17.5% underwent additional resection after the first iMRI scan, and the number of iMRI scans per patient averaged 1.18, with a surgical time of 4.5 hours; pre-resection iUS scans revealed an average of 3.8 tumor borders adjacent to sulci in 75% of patients, and 67.5% utilized intermediate resection control, leading to intraoperative procedural changes in 37.5%; iUS demonstrated a sensitivity of 46% and specificity of 96% for residual tumors compared to iMRI. | The combined use of intraoperative ultrasound (iUS) and iMRI in a full-course resection control strategy demonstrated successful implementation with favorable surgical outcomes, potentially enhancing resection control, providing additional intraoperative information for surgical planning, and offering efficiency gains by reducing the number of iMRI scans and shortening surgery time compared to iMRI-assisted glioma surgery. | Include |
| 31 | Intraoperative MRI for newly diagnosed supratentorial glioblastoma: a multicenter-registry comparative study to conventional surgery. Shah AS, Sylvester PT, Yahanda AT, et al. 2021, USA | Retrospective cohort study, n = 640 | Adult patients with newly diagnosed supratentorial glioblastoma who underwent resection | To evaluate the impact of iMRI on extent of resection (EOR) and overall survival (OS) | In a cohort with a median age of 60.0 years, achieving gross-total resection (GTR) and 100% extent of resection (EOR) through intraoperative magnetic resonance imaging (iMRI) were associated with longer overall survival, while additional resection after iMRI improved EOR and GTR rates without increasing the risk of new permanent neurological deficits. | Intraoperative magnetic resonance imaging (iMRI) correlated with improved extent of resection and gross total resection rates, serving as a significant predictor of the latter on multivariate analysis, although it did not independently predict overall survival, necessitating further evidence to ascertain its clinical benefit. | Exclude. Off-topic focus. Limited neuronavigation utilization. |
| 32 | The value of 5-aminolevulinic acid in low-grade gliomas and high-grade gliomas lacking glioblastoma imaging features: an analysis based on fluorescence, magnetic resonance imaging, 18F-fluoroethyl tyrosine positron emission tomography, and tumor molecular factors. Jaber M, Wölfer J, Ewelt C, et al. 2015, Germany | Prospective study, n = 90 | Patients harboring gliomas without typical glioblastoma imaging features. | To identify preoperative factors (i.e., age, enhancement, 18F-fluoroethyl tyrosine positron emission tomography [18F-FET PET] uptake ratios) for predicting fluorescence in gliomas without typical glioblastomas imaging features and to determine whether fluorescence will allow prediction of tumor grade or molecular characteristics | Among 166 tumors, those with contrast enhancement, larger tumor volume, and 18F-FET PET uptake ratio >1.85 were predictive of fluorescence, which was associated with WHO grade and Ki-67/MIB-1 index, but not with MGMT promoter methylation, IDH1 mutation, or 1p19q co-deletion status; notably, fluorescing grade III gliomas exhibited a higher Ki-67/MIB-1 index compared to nonfluorescing tumors, while no differences were observed in fluorescing and nonfluorescing grade II tumors. | Age, tumor volume, and 18F-FET PET uptake are factors predicting 5-ALA-induced fluorescence in gliomas without typical glioblastoma imaging features. Fluorescence was associated with an increased Ki-67/MIB-1 index and high-grade pathology. Whether fluorescence in grade II gliomas identifies a subtype with worse prognosis remains to be determined. | Exclude. Limited data on pre- and post-operative results. |
| 33 | Krieg et al,^30^ 2015, Germany | Prospective cohort study, n = 140 | Patients with supratentorial motor eloquently located HGG undergoing preoperative nTMS (2010–2014) | To investigate whether preoperative navigated transcranial magnetic stimulation (nTMS) mapping in high-grade glioma (HGG) patients leads to changes in surgical outcomes, clinical course, and survival. | Nonsurgical motor mapping using navigated transcranial magnetic stimulation (nTMS) was associated with a significantly smaller craniotomy size, reduced occurrence of residual tumor tissue and unexpected tumor residuals, shorter median inpatient stay, increased eligibility for postoperative chemotherapy, higher rates of radiotherapy, and improved 3-, 6-, and 9-months survival compared to the non-nTMS group. | HGG patients might benefit from preoperative nTMS mapping. | Include |
| 34 | Is fluorescein-guided technique able to help in resection of high-grade gliomas? Acerbi F, Broggi M, Eoli M, et al. 2014, Italy | Prospective phase II trial, n = 20 | Participants aged 18 to 75 with suspected, newly diagnosed, and untreated high-grade gliomas (HGGs), diagnosed based on brain MRI with and without contrast, and with volumetric sequences, with the inclusion criterion requiring tumor location suitable for complete resection of the contrast-enhancing area as determined by the surgeon. | To assess the safety and preliminary efficacy of a fluorescein-guided technique for the intraoperative visualization and resection of high-grade gliomas (HGGs) using a dedicated filter on the surgical microscope | The results indicated no adverse reactions to fluorescein administration, and the fluorescein-guided technique, utilizing a dedicated filter on the surgical microscope, demonstrated a high rate of complete resection of contrast-enhanced tumors in the analyzed cases. | The fluorescein-guided technique, utilizing a dedicated filter on the surgical microscope, is safe and effective in achieving a high rate of complete resection of contrast-enhanced tumors, as determined through early postoperative MRI, in patients with high-grade gliomas. | Exclude. Off-topic focus. Limited neuronavigation utilization. |
| 35 | Cordova et al,^40^ 2016, USA | Prospective phase II trial, n = 30 | Patients who had newly diagnosed or recurrent malignant gliomas suspected by MRI were eligible for FGS. The trial included all patients 18 years of age or older with normal bone marrow, renal, and liver function, KPS ≥ 60%, and able to understand and sign an informed consent document before surgery. | To evaluate the effectiveness of fluorescence-guided surgery (FGS) using 5-aminolevulinic acid (5-ALA) in improving the extent of resection (EOR) and decreasing tumor burden in glioblastomas (GBMs), while also assessing pre-operative morphological tumor metrics and their impact on resection and patient survival. | The study found that fluorescence-guided surgery (FGS) using 5-aminolevulinic acid (5-ALA) in glioblastomas (GBMs) led to a median extent of resection (EOR) of 94.3% and a residual tumor volume (RTV) of 0.821 cm3, and identified pre-operative morphological indices, specifically surface area to volume ratio and RTV, as significantly associated with overall survival. | The study supports the effectiveness of fluorescence-guided surgery (FGS) using 5-aminolevulinic acid (5-ALA) in decreasing tumor burden and prolonging survival in glioblastoma (GBM) and highlights the significance of pre-operative morphological indices in influencing both the extent of resection and patient survival. | Include |
| 36 | Della Puppa et al,^41^ 2014, Italy | Retrospective cohort study, n = 94 | Patients affected by HGG who underwent surgery guided by 5-ALA fluorescence | To assess the extent of resection, the influence of various factors (such as tumor location, size, grade, and whether it was a recurrent surgery), and the concordance between the boundaries of fluorescent tissue and those detected by neuronavigation | In patients with high-grade gliomas undergoing surgery guided by 5-ALA fluorescence, the study found that the technique consistently enabled achieving a high rate of complete tumor removal, although factors such as tumor location, size, and whether it was a recurrent surgery played a role in predicting the surgical outcome and intraoperative findings. | The study suggests that 5-ALA fluorescence in surgery for high-grade gliomas consistently facilitates achieving complete tumor removal in all cases, but it emphasizes the significance of considering patient selection, recurrent surgeries, as well as tumor location, size, and grade as predictive factors for surgical outcomes and intraoperative observations. | Include |
| 37 | Eyüpoglu et al,^25^ 2016, Germany | Prospective cohort study, n = 100 | Patients with glioblastoma | To evaluate the impact of a novel surgical technique called DiVA on the overall survival time in patients with glioblastoma | The result of the study is that surgery using the DiVA protocol resulted in a significantly longer median survival time of 18.5 months for glioblastoma patients, compared to 14 months for the CG operated according to the current gold standard in surgical neuro-oncology. | Supra‑complete glioma surgery leads to significant prolongation of overall survival time in GBM patients. | Include |
| 38 | Guiding the first biopsy in glioma patients using estimated Ki-67 maps derived from MRI: conventional versus advanced imaging. Gates EDH, Lin JS, Weinberg JS, et al. 2019, USA | Prospective study, n = 23 | Adult patients with treatment-naïve glioma | To create clinically informative graphical maps, indicating which imaging sequences are best suited for predicting Ki-67 levels in biopsy samples. | The study successfully used a random forest algorithm with four imaging inputs to more accurately predict Ki-67 expression levels in glioma biopsies from 23 patients, compared to using conventional imaging alone. | Ki-67 levels in glioma patients can be accurately predicted using advanced imaging techniques, which improve predictive accuracy over conventional imaging, and these predictions can be graphically mapped to guide biopsies, resections, and radiation therapy. | Exclude. Off-topic focus. Limited neuronavigation utilization. |
| 39 | First-in-human study of PET and optical dual-modality image-guided surgery in glioblastoma using (68)Ga-IRDye800CW-BBN. Li D, Zhang J, Chi C, et al. 2018, China | Prospective cohort study, n = 14 | Fourteen patients, predominantly male, with a median age of 47 years, including both newly diagnosed and recurrent glioblastoma multiforme cases, with varying tumor grades and a median preoperative tumor volume of 55.49 cm³. | To explore the feasibility of the dual-modality imaging tracer and intraoperative NIRF navigation system for the surgical resection of gliomas while avoiding severe neurological deficits | The study found a strong correlation between preoperative PET uptake and intraoperative NIRF signals, high sensitivity and specificity of fluorescence-guided sampling, and satisfactory safety and resection extent, with 80% progression-free survival at 6 months. | The initial study successfully showed that the novel dual-modality imaging technique is feasible for integrated pre- and intraoperative imaging, enhancing intraoperative visualization and enabling maximum safe resection of GBM. | Exclude. Off-topic focus. Limited neuronavigation utilization. |
| 40 | Combination of hand-held probe and microscopy for fluorescence guided surgery in the brain tumor marginal zone. Richter JCO, Haj-Hosseini N, Hallbeck M, Wårdell K. 2017, Sweden | Prospective study, n = 16 | Patients with suspected high-grade glioma | To introduce a fluorescence spectroscopy-based hand-held probe (HHF-probe) for tumor identification in combination with the fluorescence guided resection surgical microscope (FGR-microscope) and evaluate them in terms of diagnostic performance and practical aspects of fluorescence detection. | The fluorescence ratio measured by the probe correlated with the fluorescence intensity seen in the FGR-microscope, with the HHF-probe showing higher sensitivity in detecting fluorescence, especially in the tumor marginal zone, compared to the FGR-microscope. | The integration of the HHF-probe into the existing tumor resection routine using the FGR-microscope proved advantageous, with the probe demonstrating superior sensitivity in detecting tumor remnants, particularly beneficial in the tumor marginal zone. | Exclude. Limited postoperative data |
| 41 | A new acoustic coupling fluid with ability to reduce ultrasound imaging artefacts in brain tumour surgery-a phase I study. Unsgård G, Sagberg LM, Müller S, Selbekk T. 2019, Norway | Prospective study, n = 15 | Patients >18 years undergoing resection for glioblastoma. | To test the fluid during surgery for histopathologically proven glioblastoma to assess safety and efficacy on image quality | The operating surgeon found ACF images to be less noisy compared to those obtained with Ringer’s solution, a finding supported by blinded evaluations and pixel intensity analyses, with no postoperative complications attributed to ACF use. | Ultrasound images acquired using ACF demonstrated significantly less noise compared to those obtained with Ringer’s solution, with the rate of adverse events being comparable to those reported in similar patient groups. | Yes |
| 42 | 5-Aminolevulinic acid-induced protoporphyrin IX fluorescence as immediate intraoperative indicator to improve the safety of malignant or high-grade brain tumor diagnosis in frameless stereotactic biopsies. von Campe G, Moschopulos M, Hefti M. 2012, Austria | Prospective study, n = 13 | Patients with suspected high-grade brain tumors | To evaluate the use of 5-aminolevulinic acid (5-ALA)-induced protoporphyrin IX fluorescence in frameless stereotaxy in improving the procedure duration and yield, and thereby reducing the risk of complications. | The samples of 13 patients revealed a positive fluorescence and were histologically confirmed as malignant or high-grade brain neoplasms. four cases were fluorescence-negative, requiring frozen section confirmation and/or multiple samples. In these cases, histology was either nonspecific gliotic changes or low-grade tumors. There were no complications related to the additional use of 5-ALA. | 5-ALA fluorescence in stereotactic biopsies can increase the safety and accuracy of these procedures by reducing sampling errors and eliminating the need for multiple samples and/or frozen section verification, creating a more accurate, faster and safer procedure for cases of suspected malignant or high-grade brain tumors situated in deep or eloquent areas. | Exclude. Off-topic focus. Limited neuronavigation utilization. |

## Included Studies After Full-Text Review

|  | **Source** | **Neuronavigation system** | **Integrated imaging modality** | **Accuracy/efficacy** | **Challenges/limitation** |
| --- | --- | --- | --- | --- | --- |
| 1 | Wang et al,^43^ 2023, P.R. China | Name not reported | Sodium Fluorescein | The study group demonstrated a significantly higher Gross Total Resection Rate (GTRR) than the CG, with no notable differences in intraoperative bleeding loss or hospital stay; Karnofsky Performance Score (KPS) and National Institutes of Health Stroke Scale (NIHSS) scores declined significantly post-treatment in the SG compared to the CG, and adverse effects did not significantly differ; in terms of survival, the SG exhibited a significantly higher overall survival (OS) compared to the CG, though progression-free survival (PFS) did not show a significant difference between the two groups. | Long-term outcomes beyond the study's follow-up period may not be captured, affecting the understanding of the surgery's lasting benefits or long-term risks. |
| 2 | Incekara et al,^21^ 2021, The Netherlands | Brainlab | iUS | Eight (35%) of 23 patients in the intraoperative B-mode ultrasound group and two (8%) of 24 patients in the standard surgery group underwent complete resection (p = 0.036). Baseline characteristics, neurological outcome, functional performance, quality of life, complication rates, overall survival and progression-free survival did not differ between treatment groups (p > 0.05). | The trial was not double-blinded, which could potentially introduce bias. However, to mitigate this, the assessment of the primary outcome, which was the complete resection of the contrast-enhancing tumor, was conducted by an independent neuroradiologist who was blinded to the treatment group allocation​. |
| 3 | Picht et al,^29^ 2015, USA | Brainlab iPlan 2.0 | nTMS + IOM | Increased the extent of resection, without compromising patient safety or long-term functional outcomes in comparison to the concurrent non-TMS CG. | Potential confounding effect of general improvements in treatment quality and more aggressive resection strategies over time, which could influence the outcomes attributed to navigated transcranial magnetic stimulation (nTMS) mapping. |
| 4 | Coburger et al,^24^ 2015, Germany | Brainlab iPlan 3.0 | 5-ALA + iMRI | Gross total resection (GTR) was achieved significantly more often (100%) in the 5-ALA&iMRI group compared to iMRI alone (82%), with a significantly higher mean extent of resection (EoR) in the 5-ALA&iMRI group (99.7%) than in the iMRI-alone group (97.4%). Complication rates were not significantly different between groups (21% iMRI group, 27% 5-ALA&iMRI group), and new permanent neurological deficits (nPND) occurred in 6% in both groups. Median progression-free survival (PFS) and overall survival (OS) did not show significant differences between the two groups. | The mean extent of resection (EoR) surpassed 97% in both groups and, given the minimal difference in EoR observed in the data, no definitive conclusion can be made regarding whether a further increase in EoR contributes to improvements in patient progression-free survival and overall survival. |
| 5 | Zhang et al,^33^ 2015, China | Brainlab iPlan 2.6 | iMRI | Higher rates of extensive resection and radiological gross total resection, better language functions at 6-month follow-up, a much lower occurrence of postoperative new aphasia, and dramatically prolonged progression-free and overall survival for glioblastoma patients compared to the CG. | The main limitation is the potential impact of edema on the Diffusion Tensor Tractography (DTT) procedure, as water molecules in edematous areas may affect accurate fiber tracking. Also, surgeons in the SG may have been influenced by the certainty of intraoperative imaging, potentially leading to a more conservative approach before the first iMRI was performed; however, it was confirmed that there was no statistical difference in extent of resection (EoR) between groups before the first iMRI, indicating that surgeons were not significantly inclined to terminate surgery earlier in the iMRI group. |
| 6 | Margetis et al,^34^ 2015, USA | Brainlab | Indigo Carmine Dye | The study demonstrated successful resection of intraoperatively identified injection points in glioma patients, revealing a reproducible staining pattern with a vividly colored and demarcated sphere of stained tissue; postoperative MRI confirmed resection of all injection points, achieving a mean tumor resection extent of 97.1%, with one patient developing a postoperative brain abscess requiring additional surgical intervention on Day 16. | The challenges with interpreting iMRI signals are highlighted due to potential interference from bleeding and trauma-related artifacts, making it difficult to distinguish residual tumor. While intravenous contrast can be used, its leakage into peritumoral tissue complicates delayed, repeat imaging interpretation. Low-field iMRI systems, though compact and cost-effective, face limitations such as increased acquisition time, reduced spatial and temporal resolution, and inconsistencies with postoperative high-field imaging findings. |
| 7 | Kubben et al,^32^ 2014, Belgium | Medtronic StealthStation | iMRI | There was no statistically significant difference in median residual tumor volume or survival between the cNN and iMRI groups, and clinical performance and health-related quality of life outcomes were not significantly different, with descriptive statistics applied due to a limited sample size. | Ultra-low-field strength iMRI does not appear to be cost-effective compared to cNN, but the study acknowledges the limitation of lacking a valid endpoint for neurosurgical studies evaluating the extent of glioblastoma resection. |
| 8 | Akay et al,^38^ 2019, Turkey | Medtronic StealthStation S7 | DTI | In a cohort of 18 patients undergoing awake craniotomy for glioma resection in eloquent areas, 50% achieved gross total resection, 38.8% underwent near total resection, and 11.1% had a subtotal resection, with intraoperative neurological deterioration observed in 14 patients, leading to permanent deficits in two patients as per sixth-month postoperative neurological examinations. | Small patient population and the absence of a CG with standalone cortical-subcortical electrical stimulation (CSC) use with awake craniotomy, making it challenging to form homogeneous experimental and CGs with similar characteristics for a controlled trial. |
| 9 | Zhang et al,^31^ 2016, China | Brainlab iPlan Cranial 3.0 | iMRI + 3D 1H-MRS | The resected volume, guided by metabolic margins, exceeded the structural volume by an average of 59.48%, with minimal postoperative deficits observed (1 patient with transient language deficit), and no long-term motor deficits. In the low-grade glioma group, no tumor recurrence was observed at the 1-year follow-up, while one patient in the high-grade glioma group experienced recurrence at the 9-month follow-up. | The study identifies challenges in the integration of 3D MRS into neuronavigation, emphasizing the need for higher magnetic intensity and addressing issues related to spatial resolution, navigational system compatibility, and the potential use of other metabolites for improved tumor margin delineation, concluding that metabolic resection, as demonstrated, may offer better prognoses than structural resection alone in glioma surgery. |
| 10 | Chen et al,^35^ 2017, China | Brainlab iPlan 2.6 | iMRI | In patients undergoing iMRI-assisted surgery for insular high-grade glioma, the detection and resection of residual tumors significantly increased the median extent of resection, resulting in a smaller mean residual tumor volume, improved Karnofsky Performance Score, and better progression-free and overall survival compared to cNN surgery. | The limitations include the challenging nature of insular high-grade gliomas (HGGs) due to the lack of anatomical landmarks, limited functional information, and the risk of overlooking hidden tumor portions. Additionally, the infiltrative behavior and close association with brain vascular supply pose constraints on achieving maximal extent of resection (EOR), as reported in previous studies. The improved EOR observed in the iMRI-assisted group is attributed to the combined use of intraoperative magnetic resonance imaging (iMRI) and neuronavigation. |
| 11 | Chan et al,^39^ 2018, Hong Kong, SAR | Name not reported | iMRI | In the study, 5-ALA was used in patients with confirmed or presumed malignant glioma, resulting in positive fluorescence in 15 cases with varying intensity; the correlation between extent of resection and the duration from 5-ALA ingestion to microscope visualization was significant, and two patients experienced temporary visual field defects, while one developed hemiparesis post-surgery. | 5-ALA is potentially toxic to liver and kidneys; contraindicated in patient with fixed neurological deficit (unless with awake mapping), patient who can have the tumor only partially resected is contra-indicated for giving 5-ALA. Close proximity to the functional area or ventricle also limits the application of 5-ALA in patients. |
| 12 | Bettag et al,^28^ 2021, Germany | Brainlab VectorVision Sky | 5-ALA | In GBM patients undergoing endoscopic surgery after complete microscopic FG resection, residual fluorescent tissue undetected with the microscope was histopathologically confirmed, resulting in a mean postoperative volume of nonenhancing but fluorescent tumor tissue at 62.0 cm3 and a relative resected volume of 244.7% compared to the contrast-enhancing tumor volume (p < 0.001). | The use of endoscopic assistance is limited for tumors located in eloquent areas of the brain, as supramaximal resection in these regions can lead to neurological deficits. Therefore, the study avoided such cases.  Deeper-seated lesions, which could potentially benefit the most from endoscopic assistance due to insufficient illumination by the microscope in deep resection cavities, were excluded. This was because the removal of healthy brain tissue within the approach corridor would have biased the volume measurements. |
| 13 | Fujii et al,^36^ 2022, Japan | Brainlab Curve Dual Display™ | iMRI | The iMRI group achieved a higher gross total resection rate (73%) compared to the CG (18%), with no significant difference in transient neurological deficits or unintended reoperations; mean operating time did not differ between the groups. | Limited number of glioma patients, a mix of histological malignancy levels, and the potential influence of improved surgeon skills over time, emphasizing the need for larger cohorts to discern prognosis differences and addressing potential confounding factors. |
| 14 | Hauser et al,^26^ 2016, Switzerland | Medtronic StealthStation S7 | 5-ALA + MRI | In 13 of 14 cases, the diagnosis was glioblastoma multiforme. One lymphoma and 1 case without fluorescence were excluded. In 11 of 12 operations, residual contrast enhancement on iMRI was found after complete resection of 5-ALA fluorescent tissue. In 1 case, the iMRI enhancement was in an eloquent area and did not undergo a biopsy. The 28 biopsies of areas suspicious for tumor on iMRI in the remaining 10 cases showed tumor in 39.3%, infiltration zone in 25%, reactive central nervous system tissue in 32.1%, and normal brain in 3.6%. | Both 5-ALA fluorescence and iMRI have their own set of limitations. Common to both is the problem of clearly delineating the boundary between the infiltration zone and healthy tissue. |
| 15 | Lu et al,^37^ 2018, China | Medtronic StealthStation TRIA i7 | IMRI | The study found that the use of 3.0 T high-field intraoperative magnetic resonance imaging (iMRI) combined with multimodal neuronavigation in the resection of gliomas in eloquent brain areas resulted in a higher resection rate, improved postoperative functional outcomes, and comparable infection rates, despite a longer operative time compared to cNN surgery. | The limitations of this study include the lack of randomness and the small sample size. In addition, apart from evaluations concerning tumor recurrence, survival, and other post-operative complications, long-term follow-up data concerning the treated patients is not currently available. |
| 16 | Schatlo et al,^27^ 2015, Switzerland | Name not reported | 5-ALA + iMRI | The combination of 5-aminolevulinic acid (5-ALA) and iMRI enhances the achievement of gross total resection, but it does not significantly impact overall or progression-free survival in patients with high-grade glioma when adjusted for resection status. | The study’s limitation includes the lack of routinely assessed predictive biomarkers and the potential confounding of biologically distinct tumor subtypes, emphasizing the need for future research to identify appropriate preoperative selection criteria for the use of iMRI in high-grade glioma cases. |
| 17 | De Witt Hamer et al,^22^ 2013, France and The Netherlands | Brainlab iPlan 3.0 | iUS | Neurological outcomes were similar between teams, and the resection probability maps showed very little differential resection in both left and right-sided tumors. | The limitation of this study is the lack of prospectively applied inclusion criteria and slight variations in surgical protocols, while the challenge of detecting smaller differences in resection probability necessitates larger patient cohorts, hindered by the dependency of regional voxels and multiple testing. |
| 18 | Hou et al,^23^ 2022, China | Brainlab iPlan 3.0 | iMRI + iUS | The extent of resection was high at 95.43%, with a gross total resection rate of 72.5%, and a median residual tumor size of 6.39 cm3; 5% of patients experienced permanent neurological deficits, 17.5% underwent additional resection after the first iMRI scan, and the number of iMRI scans per patient averaged 1.18, with a surgical time of 4.5 hours; pre-resection iUS scans revealed an average of 3.8 tumor borders adjacent to sulci in 75% of patients, and 67.5% utilized intermediate resection control, leading to intraoperative procedural changes in 37.5%; iUS demonstrated a sensitivity of 46% and specificity of 96% for residual tumors compared to iMRI. | Steep learning curve associated with using intraoperative ultrasound (iUS), iMRI, and navigation systems, as well as the financial and educational costs that limit the implementation of this strategy. |
| 19 | Krieg et al,^30^ 2015, Germany | Brainlab | nTMS + PET, 5-ALA | Nonsurgical motor mapping using navigated transcranial magnetic stimulation (nTMS) was associated with a significantly smaller craniotomy size, reduced occurrence of residual tumor tissue and unexpected tumor residuals, shorter median inpatient stay, increased eligibility for postoperative chemotherapy, higher rates of radiotherapy, and improved 3-, 6-, and 9-months survival compared to the non-nTMS group. | Potential confounders such as registration and navigation errors, imprecise determination of individual resting motor threshold (rMT) |
| 20 | Cordova et al,^40^ 2016, USA | Name not reported | 5-ALA | The study found that fluorescence-guided surgery (FGS) using 5-aminolevulinic acid (5-ALA) in glioblastomas (GBMs) led to a median extent of resection (EOR) of 94.3% and a residual tumor volume (RTV) of 0.821 cm3, and identified pre-operative morphological indices, specifically surface area to volume ratio and RTV, as significantly associated with overall survival. | Absence of a CG, the small sample size, and inhomogeneity in salvage therapies after chemoradiation. |
| 21 | Della Puppa et al,^41^ 2014, Italy | Name not reported | 5-ALA | In patients with high-grade gliomas undergoing surgery guided by 5-ALA fluorescence, the study found that the technique consistently enabled achieving a high rate of complete tumor removal, although factors such as tumor location, size, and whether it was a recurrent surgery played a role in predicting the surgical outcome and intraoperative findings. | The predictive value of recurrent surgery, tumor location, size, and grade, require validation through additional research. |
| 22 | Eyüpoglu et al,^25^ 2016, Germany | BrainLab Vector Vision | 5-ALA + iMRI | The result of the study is that surgery using the DiVA protocol resulted in a significantly longer median survival time of 18.5 months for glioblastoma patients, compared to 14 months for the CG operated according to the current gold standard in surgical neuro-oncology. | Inability to completely exclude bias due to the single center nature of the study. |
| 23 | Unsgård et al,^42^ 2019, Norway | SonoWand Invite | iUS | The operating surgeon found ACF images to be less noisy compared to those obtained with Ringer’s solution, a finding supported by blinded evaluations and pixel intensity analyses, with no postoperative complications attributed to ACF use. | Balancing the attenuation of the acoustic coupling fluid (ACF) to reduce noise without causing shadowing artefacts in the ultrasound image, leading to the testing of three different concentrations of the sound attenuating component. |

## Qualitative Data Synthesis of Findings of Included Studies

|  | **Source** | **Neuronavigation system** | **Integrated imaging modality** | **Benefits/advantages** | **Challenges and limitations** |
| --- | --- | --- | --- | --- | --- |
| 1 | Wang et al,^43^ 2023, P.R. China | Name not reported | Sodium Fluorescein | **Enhanced tumor resection:** By using sodium fluorescein, surgeons could better visualize and differentiate tumor tissue from surrounding healthy brain tissue. This enhanced visibility likely contributed to more thorough and precise resection of the tumor (p3911). | **Limitations in fluorescence detection:**  Sodium fluorescein relies on the disruption of the blood-brain barrier for its effectiveness. While the blood-brain barrier is typically disrupted in gliomas, which allows sodium fluorescein to enter and accumulate in tumor tissue, this may not be uniformly the case across all areas of a glioma. Therefore, areas with intact blood-brain barriers may not fluoresce, potentially leading to incomplete visualization of the tumor. Sodium fluorescein fluoresces at 560 nm, and while this can be readily observed, the detection may not be as clear in all scenarios. The effectiveness of fluorescence-guided surgery can vary depending on factors like the intensity of fluorescence and the depth of the tumor tissue (p3911). |
| 2 | Incekara et al,^21^ 2021, The Netherlands | Brainlab | iUS | **No prolongation of surgery time:** The use of intraoperative ultrasound did not prolong surgery time. Surgeons were able to rapidly assess for residual tumor multiple times during the surgery without extending the overall surgery duration (p5).  **Enhanced complete resection rate:** The study concluded that intraoperative B-mode ultrasound enabled complete tumor resection more often than standard surgery, which is critical in maximizing the extent of glioblastoma resection during surgery (p5). | **Use of only 2-D B-mode intraoperative ultrasound:** The trial exclusively used 2-D B-mode intraoperative ultrasound imaging, without incorporating advanced ultrasound techniques. Studies have indicated that a linear array ultrasound probe is superior in detecting tumors compared to conventional probes. The challenge becomes more significant in detecting smaller residual tumor volumes (<1 cm³).  Advanced ultrasound techniques, such as contrast-enhanced ultrasound, Doppler ultrasound, and elastography, could potentially improve tumor detection during surgery. These techniques offer greater specificity in identifying residual tumors and better discrimination between different tissues, leading to sharper identification of lesion margins (p6). |
| 3 | Picht et al,^29^ 2015, USA | Brainlab iPlan 2.0 | nTMS + IOM | **Greater rate of total resections:** The addition of presurgical nTMS mapping to the clinical routine, which already incorporated preoperative fiber tractography and intraoperative neuronavigation and electrophysiology, was shown to improve surgical outcomes. This was evident in the increased extent of resection without compromising patient safety or long-term functional outcomes compared to the non-TMS CG (p540). | **Increased rate of transient deficits:** The nTMS & IOM (Intraoperative Monitoring) group experienced a significantly higher rate of transient deficits compared to the IOM-only group (26% vs. 3%). However, it’s important to note that most of these deficits (85%) resolved within 7 days, i.e., during the hospital stay. The rate of new permanent deficits was comparable between the nTMS & IOM group and the IOM-only group (13% vs. 15%). The early postoperative MRI scans did not show vascular compromise in any of the cases with new motor deficits, suggesting that these were likely due to mechanical damage to motor eloquent tissue during surgery (p539). |
| 4 | Coburger, et al,^24^ 2015, Germany | Brainlab iPlan 3.0 | 5-ALA + iMRI | **Increased extent of resection (EoR):** Combining 5-ALA and iMRI significantly increased the EoR compared to the use of iMRI alone. This was a notable finding, as a higher EoR is generally associated with better outcomes in glioblastoma surgery (p8). | **Potential risk of new permanent neurological deficits (nPND):** There is a risk that surgeons may be guided outside of the planned safe margins when using 5-ALA, leading to an increase in new permanent neurological deficits. To avoid this, surgeons might leave residual fluorescence due to anatomical uncertainties, which can be a negative predictor for progression-free survival (PFS) (p7). |
| 5 | Zhang et al,^33^ 2015, China | Brainlab iPlan 2.6 | iMRI | **Increased extent of tumor volume resection (EoR) and rate of gross total resection (rGTR):** The study group, which underwent iMRI and functional neuronavigation-guided microsurgery, demonstrated a significantly higher EoR (95.50% vs. 89.85%) and rGTR (69.60% vs. 47.70%) compared to the CG that underwent conventional navigation-guided microsurgery. This indicates a more effective and complete tumor removal using iMRI and neuronavigation (p322). | **Influence of peritumorous edema:** The study acknowledged that peritumorous edema, common in glioma cases, especially high-grade ones, could affect the diffusion tensor tractography (DTT) procedure because water molecules in the edema area may affect correct fiber tracking (p329). |
| 6 | Margetis et al,^34^ 2015, USA | Brainlab | Indigo Carmine Dye | **Enhanced accuracy in defining deep tumor margins:** By using Indigo Carmine dye in stereotactic injections, the study was able to more accurately demarcate the deep margins of intraaxial tumors. This is particularly important because deep tumor margins may not be precisely defined due to brain shift occurring during surgery (p45). | **Development of a brain abscess:** One patient developed a brain abscess on the 16th postoperative day. This occurred in the resection cavity, which had also been used for the intraoperative placement of an external ventricular drainage catheter. This adverse event led to a temporary hold on the study and the introduction of a termination rule for the study if another brain abscess developed in any subsequently enrolled patient. Investigations into the batch of Indigo Carmine used did not reveal any contamination, and the precise cause of the infection remained undetermined (p45). |
| 7 | Kubben et al,^32^ 2014, Belgium | Medtronic StealthStation | iMRI | **No significant difference in Residual Tumor Volume (RTV):** The median RTV for the cNN group was 6.5%, compared to 13% in the iMRI group. The difference was not statistically significant (P = 0.28), suggesting that iMRI did not lead to a significantly better outcome in terms of the remaining tumor volume after surgery (p53). | **Cost-effectiveness questioned:** Given the lack of significant benefits in terms of extent of resection, clinical performance, or survival, the study concluded that ultra-low-field strength iMRI might not be cost-effective compared to conventional neuronavigation (p55). |
| 8 | Akay et al,^38^ 2019, Turkey | Medtronic StealthStation S7 | DTI | **Effective resection in eloquent areas:** The study achieved a high rate of tumor resection in eloquent areas of the brain, which are regions critical for language, sensory, and motor functions. Gross total resection was performed in 50% of the patients, near-total resection in 38.9%, and subtotal resection in 11.1% (p762)**.** | **Brain shift issues:** One of the challenges addressed by using DTI-bN during awake craniotomies is minimizing errors due to brain shifts caused by intraoperative cerebrospinal fluid (CSF) loss and other volume-reducing activities during glioma resection. While DTI-bN and subcortical stimulation help reduce the margin of error related to brain shift, the study acknowledges that the position of fiber tracts changes with the positions of other brain structures, which can be a limitation (p766).  **Practical limitations of iMRI:** iMRI is sometimes not practical, especially when using the awake craniotomy method. Challenges such as the patient being awake, potential body movements, and increased operative duration can complicate the use of iMRI in these cases (p764). |
| 9 | Zhang et al,^31^ 2016, China | Brainlab iPlan Cranial 3.0 | iMRI + 3D 1H-MRS | **Enhanced tumor delineation with CNI thresholds:** By calculating and applying choline (Cho)–N-acetylaspartate (NAA) index (CNI) maps, the study was able to quantitatively analyze and compare the metabolic volumes of tumors with structural MRI volumes. The differences in volumes based on different CNI thresholds were found to be statistically significant, indicating the effectiveness of this approach in delineating tumor boundaries more accurately (p1591).  **Improved outcomes in glioma resection:** The study performed glioma resections under 3D ^1H-MRS guidance and found that the metabolic approach led to better outcomes in resection. Specifically, for both low-grade gliomas (LGGs) and high-grade gliomas (HGGs), applying optimal CNI thresholds significantly helped in achieving effective resection. Furthermore, at the 1-year follow-up, all patients showed no tumor progression, suggesting the long-term effectiveness of this approach (p1588). | **Signal quality issues near convex or ventricular systems:** The MRS signal tends to be poor when tumors are located near convex or ventricular systems. This can result in false-positive high Choline-N-acetylaspartate (Cho-NAA) index (CNI) readings in normal brain tissue and in tissue near the cranial bone, potentially leading to inaccuracies in tumor delineation (p1591).  **Technique specific to BrainLab navigational system:** The technique is mainly applicable on the BrainLab navigational system. The 3D MRS data sets do not integrate well with other navigation modalities, such as Medtronic, or third-party software. This limitation is partly due to the different scanning parameters used for anatomical slices and spectroscopy slices (p1591). |
| 10 | Chen et al,^35^ 2017, China | Brainlab iPlan 2.6 | iMRI | **Increased Extent of Resection (EOR):** The use of iMRI significantly enhanced the median EOR from 79% (ranging from 58% to 100%) to 96% (ranging from 86% to 100%), which was notably higher than the median EOR achieved with cNN (84%, ranging from 69% to 100%). This improvement in EOR is critical in glioma surgery, as a more extensive resection is often associated with better patient outcomes (p699).  **Improved Karnofsky Performance Score (KPS) and survival outcomes:** The study found that the KPS of patients in the iMRI-assisted group was significantly higher than that of the CG (90 compared to 80) at 3 months post-surgery. Additionally, the iMRI-assisted group exhibited better median progression-free survival (PFS) of 18 months (ranging from 9 to 42 months) and overall survival (OS) of 28 months (ranging from 14 to 49 months), compared to the CG’s PFS of 15 months (ranging from 3 to 32 months) and OS of 18 months (ranging from 7 to 38 months; p700). | **Risk of hemiparesis due to injury of perforating vessels:** The study acknowledges that severe complications, such as postoperative hemiparesis, were often related to the injury of perforating vessels. This risk is particularly noted in the cNN group. Such complications underscore the need for careful surgical planning and execution, especially when dealing with complex insular HGGs, to minimize the risk of damage to critical vascular structures (p704). |
| 11 | Chan et al,^39^ 2017, Hong Kong, SAR | Name not reported | 5-ALA | **Utility in supratotal resection:** The study suggests that 5-ALA can potentially aid in achieving supratotal resection, which involves removing not only the tumor but also a margin of healthy tissue around it, potentially reducing the chances of tumor recurrence (p467).  **Positive association with pathological markers:** The study found an association between the intensity of fluorescence and pathological markers such as necrotic foci, indicating that 5-ALA might be useful in assessing tumor characteristics intraoperatively (p469). | **Photosensitivity management:** Patients were required to be protected from photosensitivity in the intensive care unit for at least 24 hours after surgery. This indicates a need for special post-operative care and monitoring (p470).  **Specific time window for optimal use:** The study highlighted the importance of using 5-ALA within a specific time window (4-12 hours after ingestion) for optimal fluorescence visualization. This requirement could be seen as a limitation in terms of surgical scheduling and planning (p470). |
| 12 | Bettag et al,^28^ 2021, Germany | Brainlab VectorVision Sky | 5-ALA | **Increased Extent of Resection (EOR):** The study demonstrated a significantly increased EOR, with the overall resected tumor volume being notably larger than the preoperative contrast-enhancing tumor volume on MRI (p3). | **Limited utility in eloquent areas:** The value of endoscopic assistance is limited for tumors located in eloquent brain areas, as supramaximal resection in these regions can lead to neurological deficits (p6). |
| 13 | Fujii et al,^36^ 2022, Japan | Brainlab Curve Dual Display™ | iMRI | **Improved tumor resection rate:** Intraoperative MRI helped identify residual tumors that might not be visible during surgery, which allowed for more complete tumor removal (p273).  **Updated navigation for anatomical correction:** Intraoperative MRI allowed for the updating of navigation systems to account for anatomical changes during surgery. This was crucial because the accuracy of preoperative imaging-based navigation systems decreases due to “brain shift,” a phenomenon caused by factors such as the effect of gravity on the brain, escape of cerebrospinal fluid, brain swelling, and surgical maneuvers (p273). | **High cost:** The introduction and maintenance of iMRI are very expensive. Costs include the MRI device itself, renovation of the operating room for noise reduction and shielding and purchasing MRI-compatible instruments. Specialist staff are also required to maintain and operate the system (p273).  **Possibility of false-negative results:** There is a risk of false-negative results where residual tumors might be overlooked in intraoperative low-field MRI. This is due to the lower resolution of the low-field MRI compared to higher-field MRI systems, and the need for immediate interpretation of iMRI results to formulate a surgical strategy (p274). |
| 14 | Hauser et al,^26^ 2016, Switzerland | Medtronic StealthStation S7 | 5-ALA + iMRI | **High Complete Resection of Enhancing Tumor (CRET) rates:** The study achieved CRET in 82% of cases, which is a significant achievement compared to previous studies using either 5-ALA fluorescence or iMRI alone (p8).  **Improved detection and risk minimization:** While 5-ALA fluorescence has limitations in detecting infiltrating tumors and has a low negative predictive accuracy, iMRI can detect tumor tissue beyond the range of 5-ALA visualization. This combination of methods allows for more refined tumor detection and resection, potentially improving the efficacy of adjuvant therapeutic regimes and minimizing the risk of leaving residual tumor tissue (p8). | **Sensitivity and prediction accuracy:** 5-ALA fluorescence lacks sensitivity in defining infiltrating tumor and shows a low negative prediction accuracy in cases where tissue macroscopically appears to be a tumor. This means that even when tissue looks like a tumor under normal light, it may not always fluoresce under 5-ALA, leading to potential underestimation of the tumor’s extent (p8).  **Risk of overresection with iMRI:** Although iMRI can detect tumor tissue beyond the range of 5-ALA visualization, there is a risk of overresection. This risk, quantified at 35.7% in the study, highlights the challenge of balancing complete tumor removal with the preservation of healthy brain tissue (p8). |
| 15 | Lu et al,^37^ 2018, China | Medtronic StealthStation TRIA i7 | iMRI | **Enhanced surgical safety and efficacy:** The combination of iMRI with multimodal neuronavigation enhances the safety and efficacy of surgery. It allows surgeons to objectively determine the spatial relationship among the lesion, eloquent area, and pyramidal tract, enabling them to choose the most appropriate surgical trajectory. This approach improves the safety of the operation by allowing for the timely detection and treatment of tumor residue or new hematomas, thus avoiding postoperative complications like rebleeding and neurological deficits (p6).  **Real-time monitoring and planning:** iMRI provides real-time monitoring of the lesion resection, allowing surgeons to identify residual tumors and modify the operation plan accordingly. This capability is crucial in cases where the tumor is adjacent to vital structures like the pyramidal tract. It helps surgeons adjust to intracranial tissue shifts during the operation and modify the reconstruction of white matter fiber bundles as needed (p5). | **Prolonged operative time and increased cost:** The use of the iMRI system has disadvantages, such as prolonging the operative time by an average of approximately 30 minutes and increasing surgical costs (p5).  **Challenges in neuronavigation:** The combination of iMRI with neuronavigation improves the safety of operations, particularly in eloquent brain regions. However, it requires accurately determining the spatial relationship among the lesion, eloquent area, and pyramidal tract, and choosing the optimal operative trajectory. The process involves rebuilding images of important structures and continuously updating the operation plan during surgery. While this enhances safety, it also adds complexity to the surgical procedure and necessitates meticulous planning and execution (p5). |
| 16 | Schatlo et al,^27^ 2015, Switzerland | Name not reported | 5-ALA + iMRI | **Improved tumor resection rate:** The combination of neuronavigation with high-field iMRI and 5-ALA has been found to provide complementary information and increase the rate of GTR in this prospective study (p1564). | **Low-resolution iMRI:** The iMRI used in the study was a low-field (0.15 Tesla) system, which has a lower resolution compared to diagnostic MRI systems (1.5–3 Tesla) and newer-generation iMRI systems (greater than 1 Tesla). This lower resolution could potentially lead to fewer Gross Total Resections (GTRs) than would have been achieved with higher field strengths, and it’s unclear if this discrepancy would significantly affect outcomes (p1564). |
| 17 | De Witt Hamer et al,^22^ 2013, France and The Netherlands | Brainlab iPlan 3.0 | iUS | **Robust technique across different experience levels:** The study demonstrated that the combination of neuronavigation and imaging modalities, along with stimulation mapping, was effective regardless of the surgical team’s experience. This indicates the robustness of these techniques in glioma resection (p7). | **Resection Probability Maps (RPMs) for group analysis:** RPMs are more suited for group analysis rather than guiding individual patient surgery, which limits their applicability in personalized surgical planning (p8). |
| 18 | Hou et al,^23^ 2022, China | Brainlab iPlan 3.0 | iMRI + iUS | **Improved stability of resection control:** The combination of iUS and iMRI contributed to more stable control over the extent of tumor resection, which is a critical factor in glioma surgery (p10).  **Efficiency comparable to iMRI-only surgeries:** Despite the additional use of iUS, the surgery time did not significantly increase compared to surgeries where only iMRI was used. This suggests that the combined approach does not compromise efficiency (p12). | **Cost of the system:** The iMRI, iUS, and  navigation systems are extremely expensive. Only a few large neurosurgical centers can afford them. Adequate training and experience are obliged for successfully utilizing these techniques during surgery (p12). |
| 19 | Krieg et al,^30^ 2015, Germany | Brainlab | nTMS + PET ± 5-ALA | **Increased rate of Gross Total Resection (GTR):** The use of nTMS was associated with a higher rate of gross total resection, which is a critical factor in the effective treatment of brain tumors (p6).  **Reduction in surgery-related rate of paresis:** Patients who underwent preoperative nTMS experienced a significantly reduced rate of surgery-related paresis compared to those who did not undergo nTMS. This suggests that nTMS contributes to preserving motor function during and after surgery (p6). | **Registration and navigation errors:** The results of preoperative motor mapping by nTMS can be affected by registration and navigation errors or imprecise determination of the individual resting motor threshold (rMT). These factors can potentially lead to inaccuracies in mappingf (p9). |
| 20 | Cordova et al,^40^ 2016, USA | Name not reported | 5-ALA | **Improved Extent of Resection (EOR):** The use of 5-ALA FGS in conjunction with neuronavigation allows for the real-time intraoperative visualization of malignant tissues under fluorescence microscopy. This improved visualization facilitates more precise and comprehensive resection of the tumor (p5). | **Severe adverse event:** One severe adverse event potentially attributable to 5-ALA was observed in the study. A patient with a history of gastrointestinal perforation experienced a second perforation within 24 hours of 5-ALA administration. Surgical correction resulted in a return to baseline shortly thereafter (p6). |
| 21 | Della Puppa et al,^41^ 2014, Italy | Name not reported | 5-ALA | **Complementing neuronavigation with 5-ALA fluorescence:** There was a significant divergence between 5-ALA fluorescence and MR contrast-enhanced tumor according to neuronavigation data. In lesions larger than 9 cm³, the 5-ALA fluorescing tissue extended significantly beyond the tumor volume scheduled by contrast-enhanced tumor at MRI. This divergence highlights the advantage of 5-ALA in extending tumor resection beyond what is visible through MRI contrast enhancement. However, the study notes the importance of neurophysiological monitoring to minimize neurological deficits, as extended resection in critical areas may risk functional preservation (p6). | **Divergence between 5-ALA fluorescence and MR contrast-enhanced tumor:** This divergence is attributed to a phenomenon known as brain shift, which occurs during surgery and leads to volumetric deformations and changes in brain morphology. Brain shift causes a continuous modification of cerebral structures, resulting in a progressive inaccuracy of neuronavigation data. As neuronavigation relies on pre-operative contrast-enhanced tumor images, this inaccuracy can become problematic during surgery. The study highlights that 5-ALA-guided surgery may extend up to 6 mm beyond the MRI contrast-enhanced tumor, posing a risk to function preservation, especially in critical areas (p6). |
| 22 | Eyüpoglu et al,^25^ 2016, Germany | Brainlab Vector Vision | 5-ALA + iMRI | **Risk of neurological deterioration:** The DiVA protocol involves the resection of tissue exhibiting both distinct and vague 5-ALA fluorescence. There is a fear that extensive resection, especially of tissue showing vague fluorescence, could increase the risk of damage to functionally eloquent brain areas, leading to neurological deterioration. However, the integration of iMRI with functional neuronavigation in the DiVA protocol allows for real-time visualization of these areas, enabling more precise control over the extent of feasible resection without causing neurological deterioration (p25756). | **Intraoperative visualization challenges:** Even with the advanced techniques used in the DiVA protocol, the challenge of effectively targeting solitary orphan tumor cells that remain in Tumor Zone III continues. These residual cells can lead to tumor recurrences, highlighting the need for improved intraoperative visualization methods to target these specific tumor cell subpopulations (p25765). |
| 23 | Unsgård et al,^42^ 2019, Norway | SonoWand Invite | iUS | **Improved image quality:** The study demonstrated that the use of ACF in ultrasound imaging during brain tumor surgery resulted in images with significantly less noise compared to those obtained with Ringer’s solution, the current standard. The enhanced image quality was beneficial in identifying remaining tumor tissue in the area below the resection cavity, which is a crucial aspect of successful brain tumor surgery (p1484). | **Image quality dependent on resection cavity size:** The improvement in image quality with ACF was found to be dependent on the size of the resection cavity. This suggests that the effectiveness of ACF in enhancing ultrasound image quality may vary based on the specific surgical scenario or the stage of tumor resection (p1484). |

## Patient Characteristics Across Included Studies

| **Source** | **Research design** | **Cohort** | **Number of patients** | **Male/Female** | **Mean age** | **Glioma grade** |
| --- | --- | --- | --- | --- | --- | --- |
| Incekara et al,^21^ 2021 | Randomized controlled trial | SG | 23 | 14 male / 9 female | 62 | probable HGG (% NR) |
|  |  | CG | 24 | 14 male / 10 female | 64 | probable HGG (% NR) |
| De Witt Hamer et al,^22^ 2013 | Retrospective cohort study | Senior Team | 56 | 26 male / 30 female | 39 | HGG, LGG (% NR) |
|  |  | Junior Team | 52 | 32 male / 20 female | 41 | HGG, LGG (% NR) |
| Hou et al,^23^ 2022 | Retrospective study |  | 40 | 18 male / 22 female | 48.28 | HGG: 77.5%; LGG: 22.5% |
| Coburger et al,^24^ 2015 | Prospective cohort matched with retrospective cohort study | SG | 33 | NR | 57 | HGG: 100% |
|  |  | CG | 33 | NR | 59 | HGG: 100% |
| Eyüpoglu et al,^25^ 2016 | Prospective cohort study | SG | 30 | 18 male / 12 female | 62 | HGG: 100% |
|  |  | CG | 75 | 42 male / 33 female | 62 | HGG: 100% |
| Hauser et al,^26^ 2016 | Prospective study |  | 11 | NR | NR | HGG: 100% |
| Schatlo et al,^27^ 2015 | Retrospective cohort study | SG | 145 | 99 male / 46 female | NR | HGG: 100% |
|  |  | CG | 55 | 38 male / 17 female | NR | HGG: 100% |
| Bettag et al,^28^ 2021 | Retrospective study |  | 12 | 8 male / 4 female | 65 | HGG: 100% |
| Picht et al,^29^ 2015 | Retrospective cohort study | SG | 93 | 50 male / 43 female | 53.9 | HGG: 100% |
|  |  | CG | 34 | 19 male / 15 female | 53.2 | HGG: 100% |
| Krieg et al,^30^ 2015 | Prospective cohort study | SG | 70 | 45 male / 25 female | 58 | HGG: 100% |
|  |  | CG | 70 | 45 male / 25 female | 60.3 | HGG: 100% |
| Zhang et al,^31^ 2016 | Retrospective study |  | 15 | 7 male / 8 female | NR | HGG: 47%; LGG: 53% |
| Kubben et al,^32^ 2015 | Randomized controlled trial | SG | 7 | 4 male / 3 female | 61 | probable HGG (% NR) |
|  |  | CG | 7 | 4 male / 3 female | 66 | probable HGG (% NR) |
| Zhang et al,^33^ 2015 | Prospective cohort study | SG | 112 | 76 male / 36 female | 45.3 | HGG: 47.4%; LGG: 52.7% |
|  |  | CG | 86 | 53 male / 33 female | 42.8 | HGG: 45.4%; LGG: 54.6% |
| Margetis et al,^34^ 2015 | Prospective study |  | 10 | 6 male / 4 female | 49 | HGG: 60%; LGG: 40% |
| Chen et al,^35^ 2017 | Retrospective cohort study | SG | 51 | 32 male / 19 female | 54.2 | HGG: 100% |
|  |  | CG | 22 | 14 male / 8 female | 51.8 | HGG: 100% |
| Fujii et al,^36^ 2022 | Retrospective cohort study | SG | 11 | 4 male / 7 female | 46.7 | HGG: 55%; LGG: 45% |
|  |  | CG | 11 | 8 male / 3 female | 47.7 | HGG: 55%; LGG: 45% |
| Lu et al,^37^ 2018 | Prospective cohort study | SG | 20 | 9 male / 11 female | 44.9 | HGG: 60%; LGG: 40% |
|  |  | CG | 20 | 9 male / 11 female | 45.45 | HGG: 80%; LGG: 20% |
| Akay et al,^38^ 2019 | Retrospective study |  | 18 | 15 male / 3 female | 46 | HGG: 50%; LGG: 50% |
| Chan et al,^39^ 2017 | Retrospective study |  | 16 | 4 male / 12 female | 48.3 | HGG: 75% LGG: 18.75% |
| Cordova et al,^40^ 2016 | Prospective study |  | 30 | 20 male / 10 female | 60 | HGG: 100% |
| Della Puppa et al,^41^ 2014 | Retrospective study |  | 94 | 53 male / 41 female | 58 | HGG: 100% |
| Unsgård et al,^42^ 2019 | Prospective study |  | 15 | 11 male / 4 female | 64 | HGG: 100% |
| Wang et al,^43^ 2013 | Randomized controlled trial | SG | 60 | 31 male / 29 female | 52.11 | HGG: 100% |
|  |  | CG | 60 | 38 male / 22 female | 54.25 | HGG: 100% |

(SG: study group or intervention group; CG: control group; HGG: high-grade glioma; LGG: low-grade glioma)

## Neuronavigation and Advanced Imaging with Neurosurgical Metrics and Outcomes

| **Source** | **NS** | **Cohort** | **IAI** | **Number of patients** | **GTR** | **p value** | **EOR** | **p value** | **OT** | **p value** | **OS** | **p value** | **PFS** | **p value** |
| --- | --- | --- | --- | --- | --- | --- | --- | --- | --- | --- | --- | --- | --- | --- |
| Kubben et al,^32^ 2015 | Medtronic StealthStation | SG | NS + iMRI | 7 | NR |  | 13% (median, residual tumor volume) | 0.28 | NR |  | 13 months (median) | 0.81 | NR |  |
|  |  | CG | NS | 7 | NR |  | 6.5% (median, residual tumor volume) |  | NR |  | 15.5 months (median) |  | NR |  |
| Zhang et al,^33^ 2015 | Brainlab iPlan 2.6 | SG | NS + iMRI | 112 | 69.60% | 0.002 | 95.50% (mean) | <0.001 | NR |  | 19.6 months (median) | <0.001 | 12.5 months (median) | <0.003 |
|  |  | CG | NS | 86 | 47.70% |  | 89.85% (mean) |  | NR |  | 13 months (median) |  | 6.6 months (median) |  |
| Chen et al,^35^ 2017 | Brainlab iPlan 2.6 | CG | NS + iMRI | 51 | NR |  | 96% (median) | 0.031 | 390 min | 0.716 | 28 months (median) | 0.035 | 18 months (median) | 0.010 |
|  |  | SG | NS | 22 | NR |  | 84% (median) |  | 378 min |  | 18 months (median) |  | 15 months (median) |  |
| Fujii et al,^36^ 2022 | Brainlab Curve Dual Display™ | SG | NS + iMRI | 11 | 73% | 0.033 | NR |  | 465.8 min | 0.974 | NR |  | NR |  |
|  |  | CG | NS | 11 | 18% |  | NR |  | 483.6 min |  | NR |  | NR |  |
| Lu et al,^37^ 2018 | Medtronic StealthStation TRIA i7 | SG | NS + iMRI | 20 | 96.55% | 0.002 | NR |  | 355.85 min | 0.011 | NR |  | NR |  |
|  |  | CG | NS | 20 | 87.70% |  | NR |  | 302.45 min |  | NR |  | NR |  |
| Zhang et al,^31^ 2016 | Brainlab iPlan Cranial 3.0 |  | NS + iMRI + 3D 1H-MRS | 15 | 86.67% |  | NR |  | NR |  | NR |  | 12 months |  |
| Akay et al,^38^ 2019 | Medtronic StealthStation S7 |  | NS + iMRI (DTI) | 18 | 50% |  | NR |  | NR |  | 15.3 months (mean) |  | 36.4% (PFS at 6 months) |  |
| Incekara et al,^21^ 2021 | Brainlab | SG | NS + iUS | 23 | 35% | 0.036 | 97% (median) | 0.151 | 177 min | 0.907 | 12.4 months (median) | 0.751 | 7.5 months (median) | 0.937 |
|  |  | CG | NS | 24 | 8% |  | 95% (median) |  | 179 min |  | 12.2 months (median) |  | 7.7 months (median) |  |
| De Witt Hamer et al,^22^ 2013 | Brainlab iPlan 3.0 | Senior Team | iUS | 56 | 41% | <0.001 | 66% (median) | <0.001 | NR |  | NR |  | NR |  |
|  |  | Junior Team | NS | 52 | 73% |  | 92% (median) |  | NR |  | NR |  | NR |  |
| Unsgård et al,^42^ 2019 | SonoWand Invite |  | NS + iUS | 15 | NR |  | NR |  | NR |  | 10.9 months (median) |  | 42% (PFS at 6 months) |  |
| Hou et al,^23^ 2022 | Brainlab iPlan 3.0 |  | NS + iUS + iMRI | 40 | 72.50% |  | 95.43% (mean) |  | 270 min |  | NR |  | NR |  |
| Chan et al,^39^ 2017 | Name not reported |  | NS + iUS + 5-ALA | 16 | 56.25% |  | NR |  | 290.4 min |  | NR |  | NR |  |
| Cordova et al,^40^ 2016 | Name not reported |  | NS + 5-ALA | 30 | NR |  | 94.30% (median) |  | NR |  | 81% at 6 months, 52% at 9 months, 39% at 12 months |  | 45% at 6 months, 29% at 9 months, 23% at 12 months |  |
| Della Puppa et al,^41^ 2014 | Name not reported |  | NS + 5-ALA | 94 | 93% |  | NR |  | NR |  | NR |  | NR |  |
| Bettag et al,^28^ 2021 | Brainlab VectorVision Sky |  | NS + 5-ALA | 12 | 100% |  | 244.70% (mean) |  | NR |  | NR |  | NR |  |
| Hauser et al,^26^ 2016 | Medtronic StealthStation S7 |  | NS + 5-ALA + iMRI | 11 | 82% |  | NR |  | NR |  | 15.3 months (mean) |  | 36.4% (PFS at 6 months) |  |
| Coburger et al,^24^ 2015 | Brainlab iPlan 3.0 | SG | NS + 5-ALA + iMRI | 33 | 100% | <0.010 | 99.70% (mean) | <0.004 | NR |  | 18 months (median) | <0.718 | 6 months (median) | <0.309 |
|  |  | CG | NS + iMRI | 33 | 82% |  | 97.40% (mean) |  | NR |  | 17 months (median) |  | 6 months (median) |  |
| Eyüpoglu et al,^25^ 2016 | Brainlab VectorVision | SG | NS + 5-ALA + iMRI | 30 | 100% |  | 136% (median) |  | NR |  | 18.5 months (median) | 0.0004 | NR |  |
|  |  | CG | NS + iMRI | 75 | 100% |  | NR |  | NR |  | 14 months (median) |  | NR |  |
| Schatlo et al,^27^ 2015 | Name not reported | SG | NS + 5-ALA + iMRI | 145 | 45% | 0.035 | NR |  | NR |  | 17.9 months (median) | 0.043 | 10.6 months (median) | 0.19 |
|  |  | CG | NS + 5-ALA | 55 | 30% |  | NR |  | NR |  | 13.8 months (median) |  | 7 months (median) |  |
| Margetis et al, ^34^ 2015 | Brainlab |  | NS + Indigo Carmine dye | 10 | NR |  | 97.1% (mean) |  | NR |  | NR |  | NR |  |
| Wang et al,^43^ 2013 | Name not reported | SG | NS + Sodium Fluo-rescein | 60 | 86.67% | 0.027 | NR |  | 229.11 min | <0.001 | 11.5 months (median) | 0.013 | 9.5 months (median) | 0.079 |
|  |  | CG | NS | 60 | 60% |  | NR |  | 285.13 min |  | 9.6 months (median) |  | 7.5 months (median) |  |
| Picht et al,^29^ 2015 | Brainlab iPlan 2.0 | SG | NS+ nTMS + IOM | 93 | 61% | 0.034 | 85.40% (mean) | 0.027 | 219 min |  | NR |  | NR |  |
|  |  | CG | IOM ± NS | 34 | 45% |  | 75.90% (mean) |  | 228 min |  | NR |  | NR |  |
| Krieg et al,^30^ 2015 | Brainlab | SG | NS+ nTMS + PET ± 5-ALA | 70 | NR |  | 34.3% (rate, residual tumor tissue) | 0.0172 | 201 minutes |  | 15.7 months (mean) | 0.1310 | NR |  |
|  |  | CG | NS + PET ± 5-ALA | 70 | NR |  | 54.3% (rate, residual tumor tissue) |  | 208 minutes |  | 11.9 months (mean) | <0.718 | NR |  |

(NS: neuronavigation system; IAI: integrated advanced imaging; GTR: gross total resection rate; EOR: extent of resection; OT: operative time; OS: overall survival; PFS: progression-free survival; min: minutes)
